# Targeting mitochondrial dysfunction and apoptosis in 5-fluorouracil–induced cardiotoxicity using kaempferol-mediated selenium nanoparticles

**DOI:** 10.1038/s41598-026-69038-9

**Published:** 2026-09-12

**Authors:** Sami Mohamed Nasr, Ahmed Mahmoud Gad, Shahd Abdelsalam Taha, Nouran H. Hindam, Mai Rabea Mohamed, Aya G. Badawy, Mariam Mohamed Ali, Moustafa Mahmoud Abdelaziz, Khaled M. Alam-ElDein, Mohamed H.A. Gadelmawla

**Affiliations:** 1 School of Biotechnology, Badr University in Cairo, Badr City, 11829 Cairo Egypt; 2 Department of Biochemistry and Molecular Biology, Theodor Bilharz Research Institute, Giza, Egypt; 3 Molecular Biology and Biotechnology Department, School of Biotechnology, Badr University in Cairo, Badr City, 11829 Cairo Egypt; 4 Histology Department, School of Biotechnology, Badr University in Cairo, Badr City, 11829 Cairo Egypt

**Keywords:** Kaempferol, Cardiotoxicity, Nanoparticle, 5-FU, Apoptosis, Inflammation, Biochemistry, Cancer, Cardiology, Cell biology, Drug discovery, Medical research

## Abstract

5-Flurouracil (5-FU) is still considered as one of the most popular antimetabolite chemotherapy drugs to treat a wide variety of solid tumors. Dose limiting toxicity on major organs, especially cardiovascular system limits the therapeutic use of 5-FU. The current paper addresses the cardioprotective effect of green synthesized selenium nanoparticles through kaempferol (KMP-SeNPs) on the cardiotoxicity induced by 5-FU in a rat model. 35 male Wistar rats were divided equally into five groups, namely control, 5-FU, Na_2_SeO_3_&5-FU, KMP&5-FU, and KMP-SeNPs&5-FU. The 5-FU consumed 5 days induced cardiotoxicity and there were 21 days of oral treatment. The levels of cardiac injury markers, oxidative stress indices, antioxidant enzymes, inflammatory/anti-inflammatory cytokines and apoptotic markers were measured by ELISA, gene expression and immunohistochemistry. The evaluation of cardiac tissue was done by using histopathology. 5-FU caused significant cardiotoxicity detected by increased cardiac enzymes, oxidative stress, inflammatory cytokines, apoptosis, and extensive histopathologic damage. KMP-SeNPs treatment had a great impact on reducing myocardial injury, normalizing cardiac enzymes, decreasing lipid peroxidation and NO generation, reestablishing antioxidant defenses, and increasing Nrf2 signaling and inhibiting Keap-1 expression. The expression of TNF-α, IL-6, NF-κB, Bax, and caspase-3, were significantly decreased, whereas IL-10, FOXP3, and Bcl-2 showed a significant increase, which is evidence of the great anti-inflammatory, as well as anti-apoptotic effect of KMP-SeNPs. Immunohistochemical and histopathology results supported these molecular and biochemical findings to show approximately normal cardiac architecture. KMP-SeNPs are more effective in protecting against 5-FU-induced cardiotoxicity using antioxidant, anti-inflammatory, immunoregulatory, and anti-apoptotic activities.

## Introduction

 Fluorouracil (5-FU) is an antimetabolite chemotherapeutic agent that is widely used and its cytotoxicity is attributed to its conversion into an inhibitory thymidylate synthase metabolite, which is 5-fluoro-2-deoxyuridine monophosphate. This prevents the synthesis of thymidine nucleotides, thus pausing the replication of DNA and causing cell death in fast dividing malignant and normal proliferative tissues^1^. In spite of its therapeutic potential the clinical use of 5-FU is often limited because of its serious adverse effects most prominently cardiotoxicity. Following 5-FU, the second most cardiotoxic anticancer agent presented in the literature is cardiovascular disturbances, reported in as many as one-third of individuals treated with the drug^2^. The clinical manifestations include tachyarrhythmias, myocardial ischemia, blood pressure mal-regulation, heart failure, and cardiomyopathy^3^.

Both experimental and clinical data indicate that 5-FU undermines endothelial integrity, which results in the extravasation of plasma, inflammatory infiltration, and myofibrillar degeneration of cardiac tissue^4^. Furthermore, cellular impairment of endothelial nitric oxide synthase (NOS) activity helps to cause coronary vasospasm and subsequent heightened vasoconstrictive effect via protein kinase C-dependent mechanisms^5^. There is also one more primary role of oxidative stress because 5-FU significantly increases the levels of reactive oxygen species (ROS) and stimulates cardiomyocyte apoptosis, which exacerbates myocardial damage^1^.

Flavanol kaempferol is a naturally found flavonoid, and can be found in tea, cruciferous vegetables, berries, tomatoes and many medicinal plants. It has been extensively referred to as a primary mediator of therapeutic effects of polyphenol diets. As emphasized in cardioprotective properties of kaempferol^6^, kaempferol has a variety of pharmacodynamic effects, such as a strong antioxidant, anti-inflammatory, anti-apoptotic, anti-fibrotic and mitochondrial-stabilizing properties^7^. Considerable amounts of experimental evidence indicate that kaempferol attenuates oxidative stress through the inhibition of superfluous formation of ROS, the enhancement of endogenous antioxidant enzyme systems (SOD, CAT, GSH, GPx), and the activation of the Nrf2/HO-1 cytoprotective pathway. Simultaneously, the NF- 2 - antibiotic interaction leads to the suppression of the inflammatory reaction and, consequently, the inhibition of the expression of the main pro-inflammatory cytokines^8^.

Selenium (Se) promotes cardiovascular homeostasis by its impact into selenoproteins including glutathione peroxidase (GPx), thioredoxin reductase (TrxR), and selenoprotein P. All these selenoenzymes keep the redox systems in balance, eliminate peroxides, and ensure that the cardiac tissue is not an oxidative target. It is highly correlated with the increased risk of developing cardiomyopathy, arrhythmias, and oxidative myocardial damage^9–11^. The latest development in nanotechnology has placed selenium nanoparticles (SeNPs) in a better positioning against traditional Se preparations because it is an improved form of Selenium with regard to bioavailability, stability, and reduced toxicity. Their small particles enhance the easy penetration into cells and can be released in a continuous manner, thus enhancing their antioxidant, anti-inflammatory, and anti-apoptotic activity^12^.

Although oxidative stress and inflammatory signaling are identified as primary mediators of 5-FU-induced cardiotoxicity, the available preventive measures mostly are based on empirical evidence and are not mechanistic. Even though kaempferol and selenium have a cardioprotective effect by themselves, it is not clear whether nano-functionalization can enjoy a synergistic effect on cardioprotective effect or whether it will only have an additive effect on antioxidant functions. No such study has ever directly compared biosynthesized selenium nanoparticles mediated by kaempferol, with their parent compounds in a standardized 5-FU cardiotoxicity model and probed coordinated modulation of Nrf2/Keap-1, NF- kB, and apoptosis. Thus, the therapeutic significance and mechanistic benefit of KMP-SeNPs compared to conventional preparations are not determined.

Based on these characteristics, this paper examines the cardioprotective effect of kaempferol-mediated green synthesized selenium nanoparticles (KMP-SeNPs) in the cardiotoxicity provoked by 5-FU in an animal model. This study will conduct bio-chemical, molecular, and histological analyses in understanding the protective effects of KMP-SeNPs against those of kaempferol and sodium selenite individually, as well as synergistically in alleviating oxidative, inflammatory, and apoptotic injuries occurring in the heart.

## Materials and methods

### Docking

#### Software and computational environment

The Schrodinger Suite 2021 (Maestro, version 12.8) was used to perform all the molecular docking and visualization processes. It is this integrated platform that was used to carry out the process of protein and ligand preparation, receptor grid generation, molecular docking simulations and the resultant analysis of protein-ligand interactions. All computational s6teps were implemented with default parameters suggested by the software, unless indicated differently, and this guarantees methodological reproducibility and consistency.

#### Protein selection and preparation

The docking analysis was done on three major protein targets related to inflammatory signaling and response to oxidative stress inducible nitric oxide synthase (iNOS; PDB ID: 1DD7), glutathione S-transferase (GST; PDB ID: 1EV4) and tumor necrosis factor-alpha (TNF-α 01PDB ID: 2AZ5). The high-resolution crystal structures were obtained in the Protein Data Bank (PDB).

The steps involved in protein structures preparation were assignment of proper bond orders, addition of missing hydrogen atoms, correction of ionization and protonation, elimination of non-essential crystallographic water molecules, and optimization of the hydrogen-bonding network. The minimum of energy was then restrained by the means of OPLS3e force field to eliminate any steric clash and enhance structural stability. When the heavy-atom root mean square deviation (RMSD) stabilized at 0.3 A, the minimization was terminated hence well-refined protein conformations that could be used in molecular docking studies.

#### Ligand preparation

Kaempferol chemical structure was obtained in PubChem database and then prepared in LigPrep module in Maestro. The preparation of the ligands was done through energy minimization and the creation of the corresponding ionization and protonation states of the ligand at different physiological pH. These procedures guaranteed a dynamically advantageous and chemically proper ligand conformational position to be utilized in future molecular docking research.

#### Receptor grid generation

Each protein target of interest was then built as a grid receptor with the Receptor Grid Generation module of Maestro. Around the active or binding sites grid boxes were placed based on reported key amino acid residues or the position of the co-crystallized ligands if they were present. The common grid dimensions were used so that there were enough spatial coverage and conformational flexibility when docking of the ligands.

#### Molecular docking

The simulated molecules of kaempferol in the docking approach with iNOS, GST and TNF-α were performed on the Glide module in Extra Precision (XP) mode. The docking protocol enabled complete conformational freedom of the ligand whereas the protein receptors were held in a fixed form. The resulting binding poses were ranked, and assessed on the basis of Glide docking scores that give a measure of binding affinity, taking into consideration key interaction parameters.

#### Docking protocol validation and comparative docking

The reliability of the molecular docking protocol was evaluated using a re-docking approach. For each protein target, the corresponding co-crystallized ligand was extracted and subsequently re-docked into its original binding site using the same receptor-grid dimensions, force-field parameters, and Glide XP docking settings employed for kaempferol. The top-ranked re-docked pose was compared with the experimentally determined crystallographic pose, and the heavy-atom root mean square deviation (RMSD) was calculated. An RMSD value below 2.0 Å was considered indicative of successful reproduction of the crystallographic binding orientation. Established inhibitors of iNOS, GST, and TNF-α were also selected for comparative docking under identical conditions, and their Glide docking scores, binding orientations, hydrogen-bond interactions, and contacts with key binding-site residues were compared with those of kaempferol.

### Drugs

Kaempferol (CAS 520-18−3) was purchased at Sigma-Aldrich and used without any additional purification. KMP-selenium nanoparticles (KMP-SeNPs) were prepared using a green bioreduction process through the combination of solution of sodium selenite (10mM) and KMP (3.5 mg/mL) in equal ratios (12 h) and stirred. KMP acted as providing reduction and stabilization functions agent by facilitating the decrease in selenite ions to elemental selenium as well as inhibiting the aggregation of nanoparticles. The emergence of reddish color was a sign of the formation of nanoparticles. The freeze-dried product was then a colloidal suspension (FreeZone 4.5 L lyophilizer, Labconco) to get a dry KMP-SeNPs powder to be characterized and biologically studied.

### Characterization of nanoparticle

Electrophoretic light scattering method and dynamic light scattering (DLS) of the hydrodynamic particle size distribution and surface charge (zeta potential) of selenium nanoparticles mediated by kaempferol were examined, respectively. The measurements of values were done through a Zetasizer (Malvern Instruments, UK) at 25 o C at the Nano Center, Faculty of Engineering, Capital University, Egypt. Before analysis, freshly prepared KMP-SeNPs suspensions were suitably diluted with deionized water to reduce multi-scattering effects and give maximum accuracy of measurement. The particle size was given as the Z-average diameter and polydispersivity index (PDI) and the zeta potential values were measured using electrophoretic mobility and reported in millivolts (mV) to indicate the value of colloidal stability.

The morphological features and particle size of SeNPs mediated by kaempferol (KMP-SeNPs) were determined using the high-resolution transmission electron microscopy (TEM; JEOL Ltd., Tokyo, Japan). The samples were made by dropping the nanoparticle suspension on carbon–coated copper grid and letting them air dry at room temperature. TEM imaging was performed at Al-Azhar university in Cairo, Egypt, under the correct accelerating voltage conditions to come up with high resolution micrographs. The images acquired were applied to evaluate the shape of the particles, their size distribution, and their level of dispersion of the created nanoparticles.

### Experimental animals

The Vacsera Animal House, Helwan, Cairo, Egypt, was used to obtain thirty-five healthy adults male Wistar albino rats, whose body weights ranged between 120 and 150 g. After arrival, the animals were put in well-ventilated wire mesh polypropylene cages and kept under stringent laboratory conditions. The environmental parameters were kept constant to contain 12 h alternating light and dark photoperiod and ambient temperature maintained at 25 ± 2 °C. During experimental time, the animals were allowed to have free access to a commercially available normal rodent pellet food diet, and drinking water. Before the experimental procedures began, all rats were left to undergo an acclimatization period of 2 weeks so that they could be accustomed to the conditions of their housing and reduce variability that could arise because of stress.

### Experimental design

The experimental animals were assigned arbitrarily into 5 groups, with each group included seven rats. This grouping strategy was employed to systematically assess the cardioprotective potential of the investigated compounds in a model of cardiotoxicity induced by 5-FU.

Group I (CON; *n* = 7): Rats received normal saline (0.9% NaCl) orally throughout the experimental period and intraperitoneal (i.p) saline injections on the corresponding days of 5-FU administration, serving as a negative control.

Group II (5-FU; *n* = 7): Rats received 5-fluorouracil dissolved in normal saline intraperitoneally at a dose of 30 mg/kg once daily for five consecutive days (days 17–21), following the protocol of^13,14^, to induce cardiac toxicity.

Group III (Na2SeO3&5-FU; *n* = 7): Rats received sodium selenite dissolved in deionized water as source for selenium orally at a dose of 0.5 mg/kg per day for 21 consecutive days^11^, During the final five days (days 17–21), 5-FU was co-administered intraperitoneally^13^.

Group IV (KMP&5-FU; *n* = 7): Rats were treated orally with Kaempferol was initially dissolved in DMSO and subsequently diluted with deionized water under continuous stirring and bath sonication to obtain a uniform dosing dispersion Kaempferol (KMP) at a dose of 100 mg/kg per day dissolved in for 21 consecutive days^15^. During the final five days (days 17–21), 5-FU was co-administered intraperitoneally^13^.

Group V (KMP-SeNPs&5-FU; *n* = 7): Rats were treated orally with Selenium Nanoparticles biosynthesized using Kaempferol (KMP) at a dose of 0.5 mg/kg on deionized water per day for 21 consecutive days^16^. During the final five days (days 17–21), 5-FU was co-administered intraperitoneally^13^.

Rats received Kaempferol 1 h prior to 5-FU injection on the last 5 days of the experiment.

All the animals were observed in a clinical routine to evaluate the general health state and to detect the incidents of distress or other adverse effects of the treatment throughout the course of the experiment. Following the experimental period, the rats were fully anesthetized via an intraperitoneal injection of 1 mL of a ketamine–xylazine anesthetic mixture, consisting of 0.7 mL ketamine and 0.3 mL xylazine. Once sufficient anesthesia was confirmed, the animals were humanely euthanized by decapitation using a sterile surgical instrument. Rats were euthanized after one day following the last administration of the final dose to prevent the confounding of acute pharmacodynamic variability. Blood samples were obtained through the cardiac puncture followed by clotting in controlled conditions and serum was obtained by centrifugation to allow analysis of blood biochemical profiles. The cardiac tissues were immediately removed and washed twice with an ice cold 50 mM Tris-HCl buffer (pH 7.4) to remove any traces of blood and maintain the integrity of the tissues.

Systematic allotment of each heart into 3 parts then was done to carry out extensive downstream analyses. The initial portion was correctly weighed and homogenized in chilled tris homogenate buffer to give a 10% (w/v) tissue homogenate that was centrifuged at 3000 RPM at 4 °C and the supernatant thus obtained was used in biochemical tests. The second section was snap-frozen and kept at −80 °C to preserve the molecular stability of the sample in the following molecular studies, and the third section was fixed in formalin and allowed to undergo histopathological examination. All experimental methods were conducted in accordance with internationally accepted conditions of ethical regulations and were reviewed and accepted with the IACUC of Badr University in Cairo (BUC-IACUC) under the approval number, BUC-IACUC/BIO/130/A/2026. The research procedures involving animals were conducted according to the ARRIVE requirements, were guided by the UK Animals Act of 1986, and were approved by the Research Ethics Committee. All attempts were undertaken to keep the suffering of animals to the minimal level as well as to decrease the use of animals without interfering with the scientific integrity and reproducibility of the study.

### Serum cardiac enzyme analysis

The biomarkers cardiac injury were measured using the enzyme-linked immunosorbent assays (ELISA). The activity of the cardiac tissue homogenates of Lactate dehydrogenase (LDH) was determined using a commercial ELISA kit (Wuhan EIAab Science Co., China; Cat. No. E1864r). The levels of creatine kinase–myocardial band (CK-MB) were also evaluated using a certain ELISA kit (Abcam, UK; Cat. No. ab187396) in accordance with the instructions provided by its manufacturers.

### Oxidative stress and redox regulatory markers assessment

The status of oxidative stress was measured under the regard of the existing spectrophotometric and immunoassay-based tests. The extent of lipid peroxidation was determined by the amount of malondialdehyde, which is an index of oxidative damage to the membrane. The method of measuring nitrosative stress involved the measurement of nitric oxide (NO) levels by measuring the stable end products. Furthermore, the levels of Keap-1 were measured as a redox-sensitive regulatory protein in the regulation of oxidative stress and antioxidant signaling pathways.

### Antioxidant defense and redox signaling markers assessment

The endogenous antioxidant capacity was determined by establishing the reduced glutathione (GSH) levels by the DTNB colorimetric method. Enzymatic antioxidant defense was evaluated by measuring the activity of SOD, CAT, GPx, and glutathione reductase (GR) using the protocol of the standard assays and gave an idea about the efficiency of detoxification of the reactive oxygen species, as well as the efficiency of glutathione recycling. Moreover, the levels of Nrf2 were the transcriptional regulator of antioxidant and cytoprotective marker.

### Pro-inflammatory markers evaluation

The measures of pro-inflammatory responses in tissue homogenates have been performed with the help of standardized immunoassay techniques. ELISA kits were used to measure TNF-α and IL-6 values as the indicators of inflammatory activation and cytokine mediated signaling according to the directions of the manufacturers.

###  Anti-inflammatory markers evaluation

Another test done was the determination of IL-10 levels in tissue homogenates using specific ELISA kits at the protocols of the manufacturers to determine the level of anti-inflammatory and immunoregulatory status. The IL-10 was quantified as one of the key cytokines that inhibit the pro-inflammatory signaling and reduce the tissue inflammatory injury. Assays were performed under standardized conditions of analysis to give credible information on the endogenous counter-regulatory processes that are activated in case of tissue injury^17,18^.

### Pro-apoptotic markers assessment

The concentrations of the pro-apoptotic proteins Bax and caspase-3 were measured in cardiac tissue homogenates using commercially available rat-specific ELISA kits obtained from MyBioSource (San Diego, CA, USA). Bax was quantified using Cat. No. MBS175205, whereas caspase-3 was determined using Cat. No. MBS175203. All assays were performed according to the manufacturer’s instructions to evaluate activation of the apoptotic pathway in cardiac tissue.

### Anti-apoptotic marker assessment

The anti-apoptotic response was assessed by measuring Bcl-2 concentrations in cardiac tissue homogenates using a rat-specific ELISA kit purchased from MyBioSource (San Diego, CA, USA; Cat. No. MBS175204). The assay was conducted in accordance with the manufacturer’s protocol to quantify this key cell-survival protein.

### Gene expression analysis of NF-κB, and FOXp3

Total RNA was isolated from kidney tissue using TRIzol reagent (Life Technologies, MD, USA according to the manufacturer’s instructions. RNA concentration and purity were determined spectrophotometrically by measuring the absorbance at 260 and 280 nm, and samples with an A260/A280 ratio of 1.8–2.0 were used for subsequent analyses. Complementary DNA (cDNA) was synthesized from 1 µg of total RNA using MultiScribe™ Reverse Transcriptase Kit (Applied Biosystems, CA, USA) following the manufacturer’s protocol.

Quantitative real-time PCR was performed using Power SYBR™ Green PCR Master Mix (Applied Biosystems, Foster City, CA, USA) on an Applied Biosystems 7500 platform. The primer sequences used for amplification of NF-κB, and FOXp3 and housekeeping gene (β-actin) are listed in Table 1. The amplification protocol consisted of an initial denaturation at 95 °C for 10 min, followed by 40 cycles of denaturation at 95 °C for 15 s and annealing/extension at 60 °C for 60 s. At the end of the amplification cycles, a melt-curve analysis (60–95 °C) was performed to confirm amplification specificity by verifying the presence of a single melting peak and the absence of nonspecific amplification or primer-dimer formation. Each qRT-PCR run included no-template controls (NTCs) and no-reverse-transcriptase (no-RT) controls to exclude reagent contamination and genomic DNA amplification, respectively.

Relative gene expression was calculated using the 2^−ΔΔCt^ method. The threshold cycle (Ct) values of the target genes were normalized to the housekeeping gene β-actin to obtain ΔCt values (ΔCt = Ct-target – Ct-β-actin). The ΔΔCt values were calculated relative to the control group, and the relative expression levels were expressed as 2^−ΔΔCt^. Each sample was analyzed in triplicate, and the mean Ct value was used for statistical analysis^19,20^.

**Table 1:** Primers sequences of genes analyzed in qRT-PCR.

Target	Forward primer		Reverse primer
β. actin	TCC TCC TGA GCG CAA GTA CTCT	GCT CAG TAA CAG TCC GCC TAGAA	
NF-κB	GCA AAC CTG GGA ATA CTT CAT GTG ACT AAG	ATA GGC AAG GTC AGA ATG CAC CAA AGTCC	
FOXP3	CACCTGGCTGGGAAAATGG	GGAGCCCTTGTCGGATGA	

### Immunohistochemical evaluation of Keap-1 and Nrf-2 in cardiac tissue

The spatial distribution patterns of the Keap-1 and Nrf-2 were measured using immunohistochemistry in the cardiac sections. Heart tissues which are microtomed in paraffin were pre-incubated with hydrogen peroxide to neutralize endogenous peroxidase activity followed by the microtending the samples into sections of 4 to 5 5 mm. The sections were then left overnight at 4 o C to rabbit polyclonal primary antibodies that targeted Keap-1 and Nrf-2. Biotinylated goat anti-rabbit IgG was then added to the slides, which were then rinsed with phosphate-buffered saline (PBS) and then at 30 C was provided with streptavidin-peroxidase detection system after 30 min. It was visualized by using the DAB technique to visualize antigen-antibody complexes to give a characteristic brown precipitate and counterstained using hematoxylin to define nuclear architecture. A Leica DM3000 microscope was used at 400 magnifications to record patterns of staining and intensity of signal. ImageJ software (NIH, USA) was used to perform quantitative analysis in which mean optical density (MOD) values of various randomly chosen fields per section were obtained which gave an objective measure of Keap-1 and Nrf-2 immuno-expression^21,22^.

### Histopathological examination

A 24-hour formaldehyde fixed 24 h cardiac tissues was subjected to a graded ethanol series (70–100%) to dehydrate the tissues. This was done by clearing them with xylene and embedding the specimens in paraffin to create stable tissue blocks that could be sectioned. Using a rotary microtome, thin sections approximately 5 μm thick were prepared and subsequently stained with hematoxylin and eosin (H&E) to visualize overall myocardial architecture and cellular morphology. Prepared slides were mounted and assessed under a light microscope (Leica DM3000 microscope, Germany) for detailed histopathological assessment^23,24^.

### Statistical analysis

All the quantitative data were displayed as mean and standard error of mean (SEM) which were compared using GraphPad Prism software (version 21.0, San Diego, CA, USA). The intergroup comparisons were undertaken by using one-way analysis of variance (ANOVA) to determine the overall statistical differences among the experimental groups. When it was found that there were significant main effects, a post hoc test was used, which was the multiple range test of Duncan. *P* < 0.05 taken as significant, which indicated the existence of meaningful effects of the treatment.

## Result

### In silico analysis

#### Validation of the molecular docking protocol

**Fig. 1:**
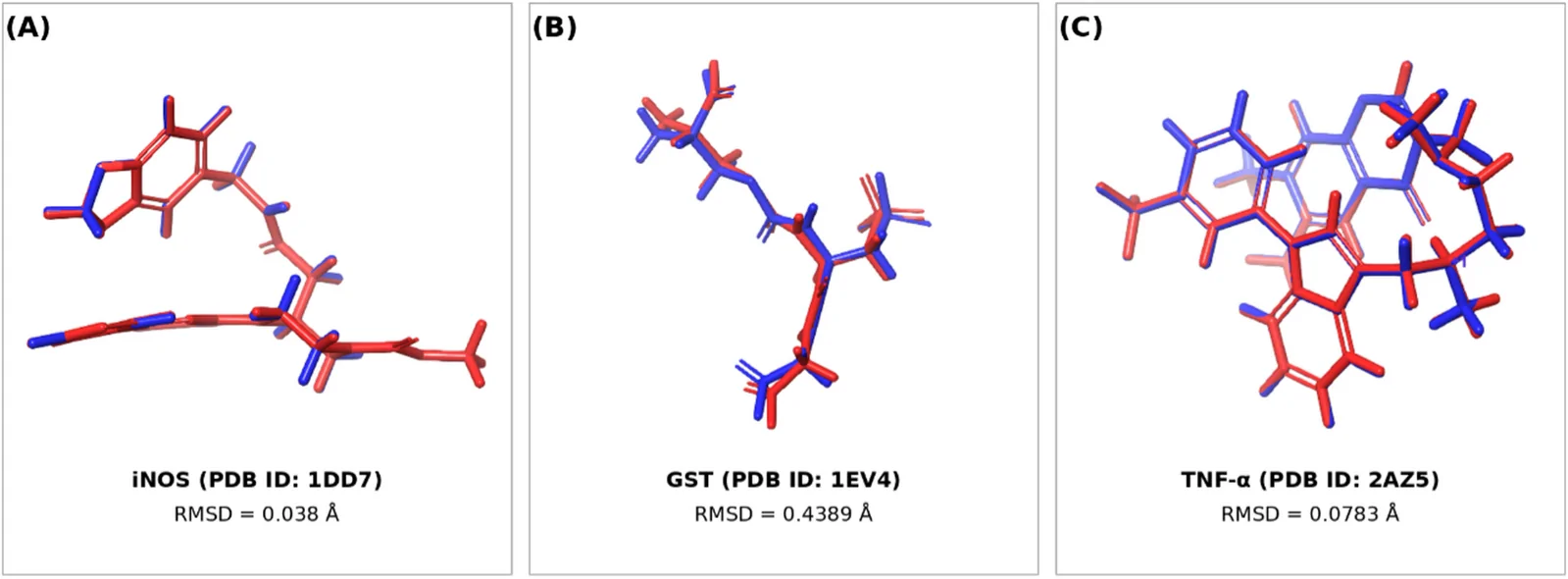
Validation of the molecular docking protocol by re-docking analysis. Superposition of the crystallographic ligand poses (red) and their corresponding top-ranked re-docked poses (blue) within the binding sites of (A) iNOS, (B) GST, and (C) TNF-α. The calculated heavy-atom RMSD values were 0.038, 0.4389, and 0.0783 Å, respectively.

Re-docking of the corresponding co-crystallized ligands into the original binding sites of iNOS, GST, and TNF-α reproduced their experimentally determined binding orientations. The calculated heavy-atom RMSD values between the crystallographic and re-docked ligand poses were 0.0380 Å for iNOS, 0.4389 Å for GST, and 0.0783 Å for TNF-α. All RMSD values were markedly below the accepted threshold of 2.0 Å, indicating close agreement between the experimental and predicted ligand poses and supporting the reliability of the applied Glide XP docking protocol. Visual superposition of the crystallographic and re-docked poses further demonstrated close spatial overlap within the respective binding pockets (Fig. 1).

#### Docking of kaempferol with inducible nitric oxide synthase (iNOS)

**Fig. 2:**
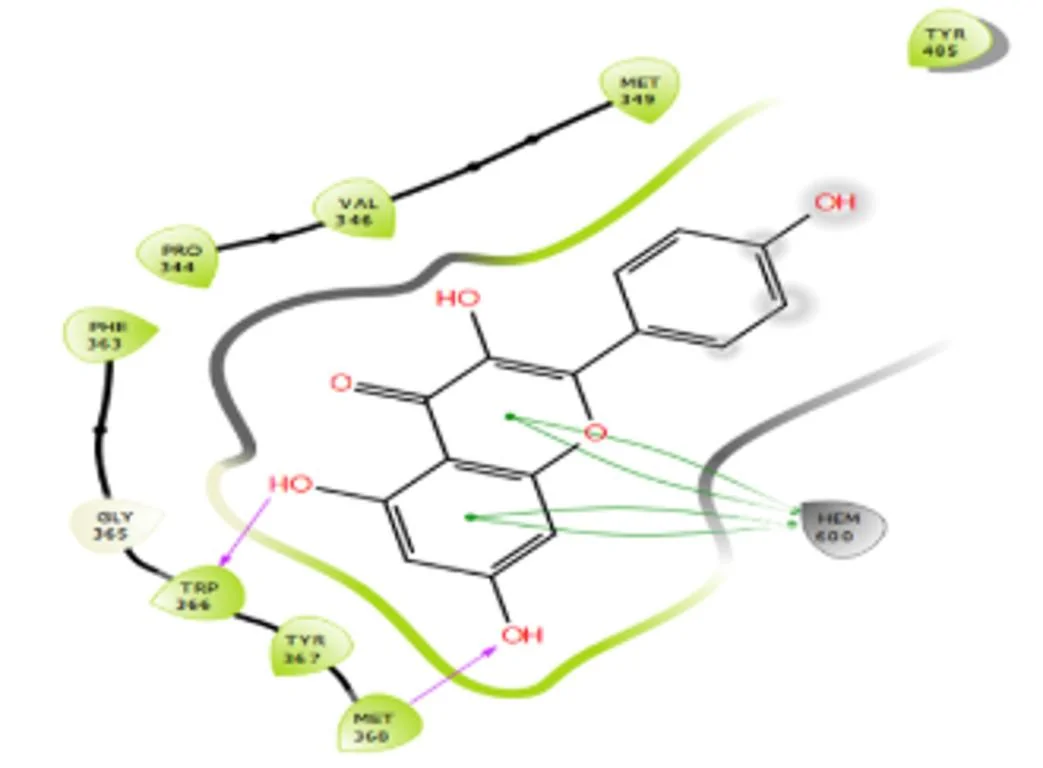
Two-dimensional interaction diagram of Kaempferol docked into the active site of iNOS (PDB ID: 1DD7).

Kaempferol demonstrated a high binding affinity toward inducible nitric oxide synthase (iNOS; PDB ID: 1DD7), as reflected by a favorable Glide docking score of − 9.199 kcal/mol. The compound was effectively positioned within the enzyme’s active site, where it established several stabilizing interactions. Notably, kaempferol acted as a hydrogen bond donor to TRP366 and as a hydrogen bond acceptor to MET368. The presence of these interactions supports the formation of a stable protein–ligand complex and suggests a potential inhibitory role of kaempferol against iNOS enzymatic activity **(**Fig. 2**).**

#### Docking of kaempferol with glutathione S-transferase (GST)

**Fig. 3:**
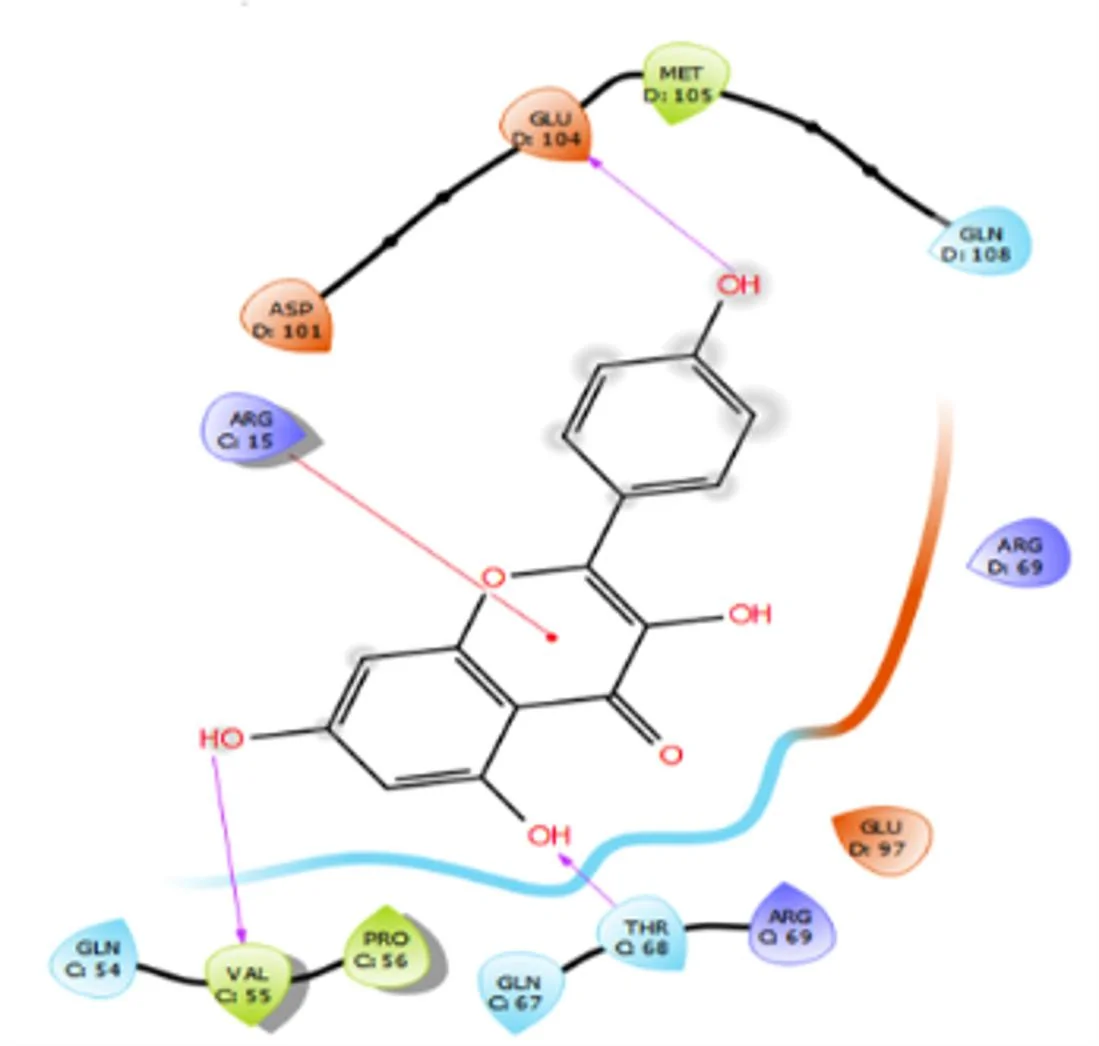
Two-dimensional interaction diagram of Kaempferol docked into the binding site of GST (PDB ID: 1EV4).

Docking simulations of kaempferol with glutathione S-transferase (GST; PDB ID: 1EV4) yielded a Glide docking score of − 5.691 kcal/mol, reflecting a moderate binding affinity. Within the GST binding pocket, kaempferol established hydrogen bond donor interactions with Val55 and Glu104, hydrogen bond acceptor interactions with Thr68, as well as π–cation interactions with Arg15. Although the predicted binding strength was lower than that observed for iNOS and TNF-α, the presence of these stabilizing contacts suggests that kaempferol may exert a modulatory influence on GST function, potentially impacting GST-mediated detoxification processes **(**Fig. 3**).**

#### Docking of kaempferol with TNF-α

**Fig. 4:**
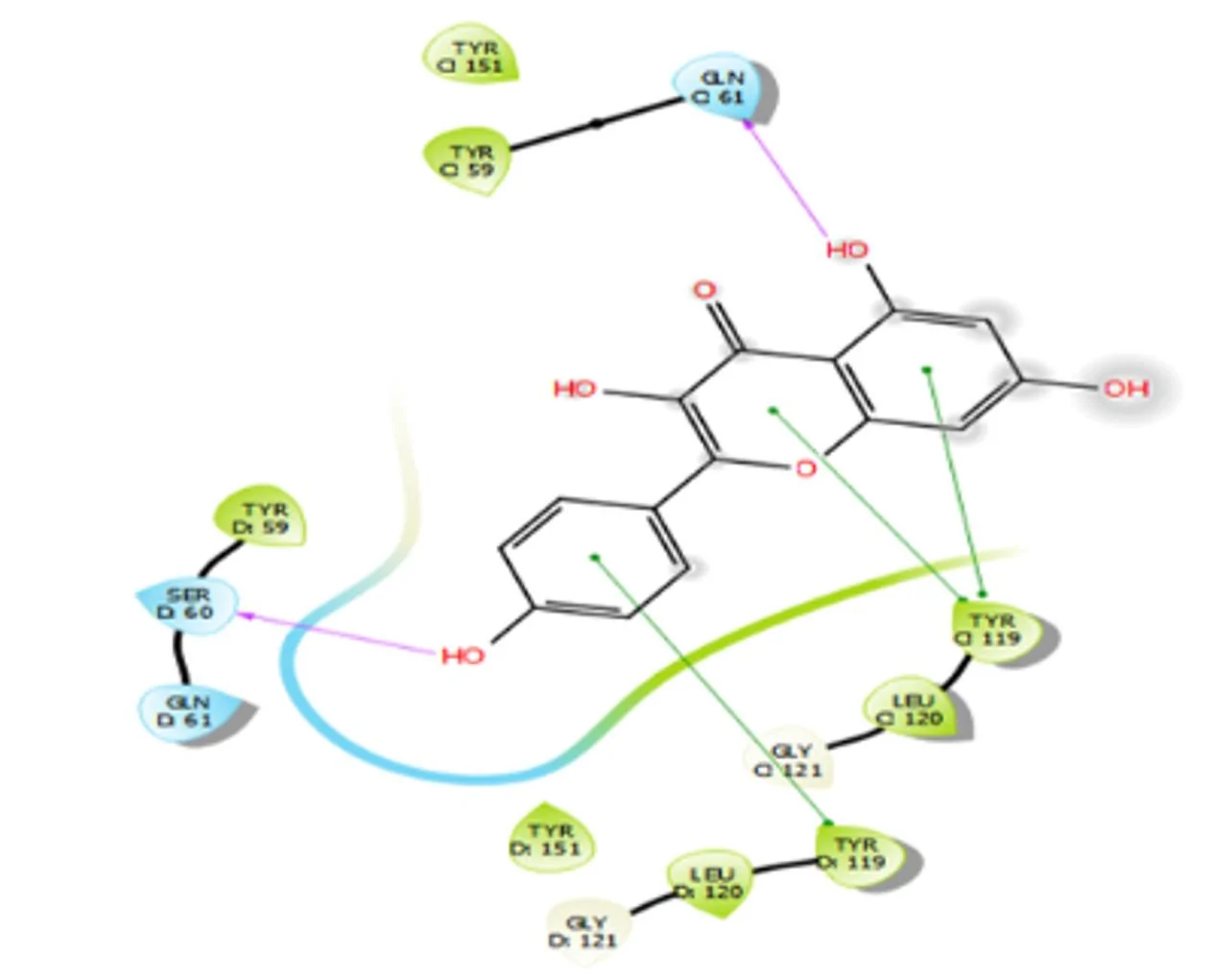
Two-dimensional interaction diagram of Kaempferol docked into the TNF-α binding site (PDB ID: 2AZ5).

The most pronounced interaction was observed between kaempferol and TNF-α, as indicated by a favorable Glide docking score of − 6.820 kcal/mol. Kaempferol was positioned stably within the TNF-α binding region, forming two hydrogen bond donor interactions with key residues, namely Gln61 and Ser60, in addition to a π–π stacking interaction with Tyr119. Collectively, these interactions support the formation of a stable protein–ligand complex and suggest a potential inhibitory effect of kaempferol on TNF-α biological activity **(**Fig. 4**)**.

###  Physicochemical characteristics of KMP-SeNPs

**Fig. 5:**
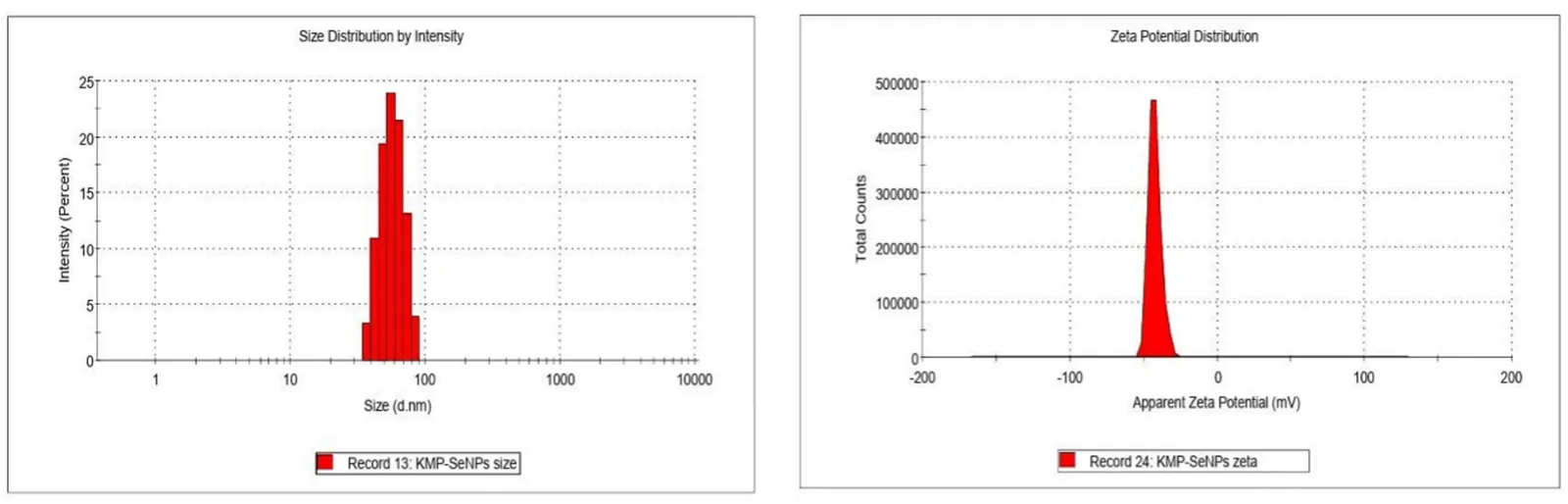
DLS and zeta potential Description of SeNPs biosynthesized using kaempferol (KMP-SeNPs). (A) particle size distribution by intensity indicating nanoscale size and good dispersion, and (B) Zeta potential distribution showing a highly negative surface charge confirming colloidal stability of KMP-SeNPs.

The dynamic light scattering analysis was used to determine the hydrodynamic size features of selenium nanoparticles synthesized with kaempferol (KMP-SeNPs). The nanoparticles had a Z-average diameter of 78.5 nm, which is indicative of a homogeneous size distribution in the nanoscale size range. The particle size profile as shown by the intensity proved to have a single, sharp peak at a value of around 80 nm, which suggests that the majority of the consistently scattered nanoparticles with a narrow distribution were present. The general quality of the measurement was good, and this proved the reliability of the size distribution data as in Fig. 5.

Surfaces charge measurement also showed that KMP-SeNPs had a significantly negative zeta potential, having the mean value of −42.5 mV and a standard deviation of 4.10 mV. A high negative surface potential is evidence of strong electrostatic repulsive forces between nanoparticles which provide great colloidal stability and particle agglomeration resistance. Together with the other physicochemical characteristics, all of them indicate that the process of nanosized selenium particle green synthesis via kaempferol has been successful and that these particles can be used in further biological and therapeutic studies.

#### Transmission electron microscopy (TEM) analysis of KMP-SeNPs

**Fig. 6:**
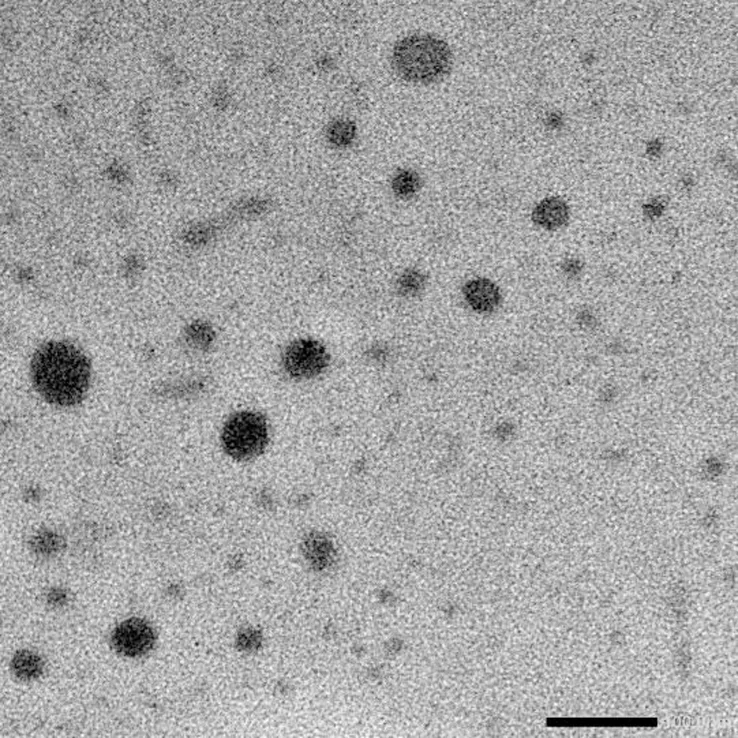
TEM image of SeNPs biosynthesized using kaempferol (KMP-SeNPs) (scale bar = 100 nm).

TEM was used to explain the morphological and size properties of selenium nanoparticles that were biosynthesized using kaempferol (KMP-SeNPs). The TEM micrograph shows that the nanoparticles are mostly spherical with clear and smooth edges and dark appearance, which proves the successful development of elemental selenium in nanoscale. These particles are extensively dispersed across the field of vision with little aggregation, which constitutes a good stabilization of kaempferol in the process of synthesizing green as in Fig. 6.

The majority of the nanoparticles are observed to have a narrow nanoscale size range, which can be well compared with the size of the particles observed in dynamic light scattering analysis. In another colloidal stability of KMP-SeNPs, the lack of big aggregates and the equal scattering of the sample also testify.

### Cardiac enzymes

**Fig. 7:**
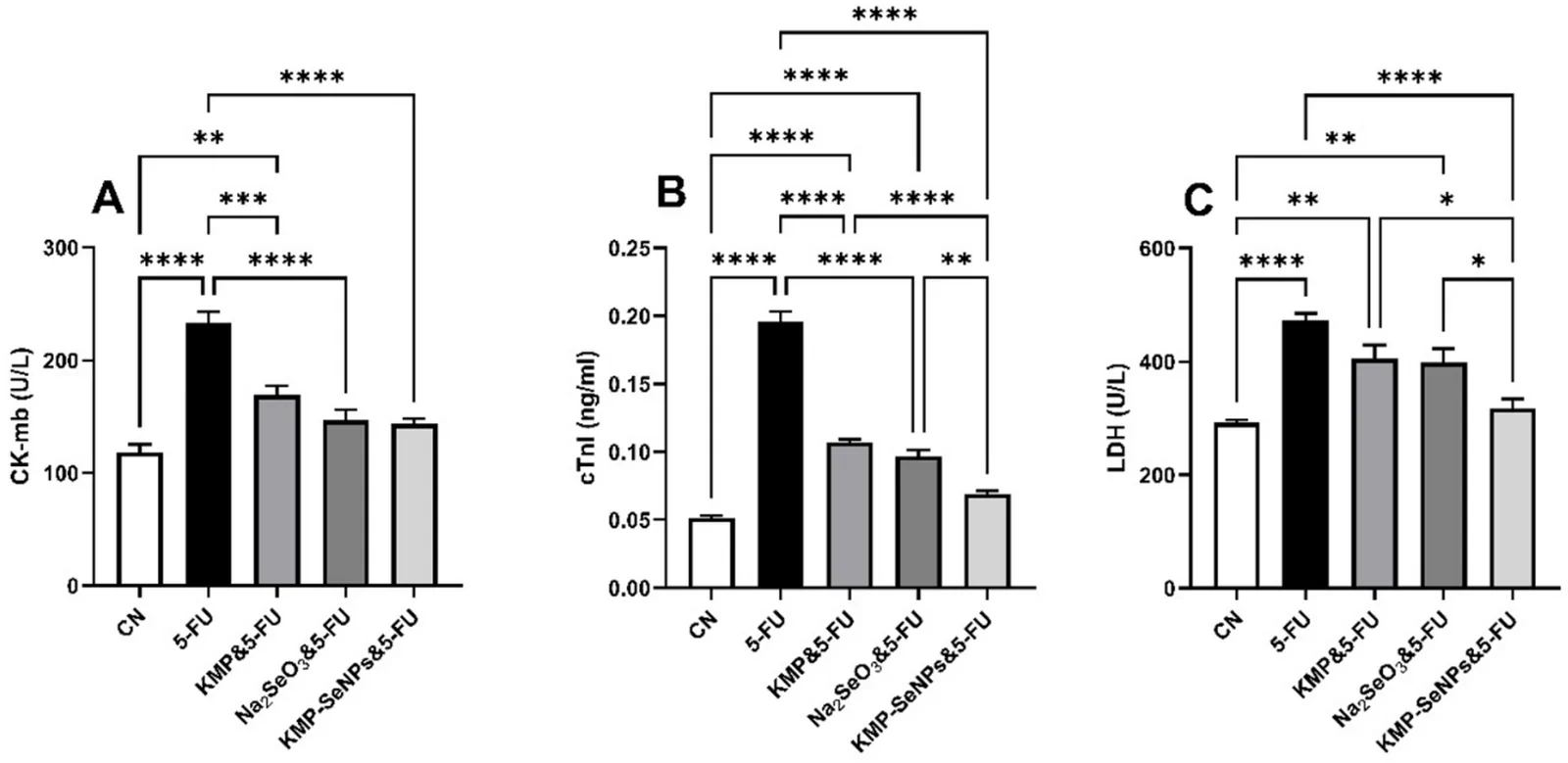
Impact of kaempferol and kaempferol-mediated selenium nanoparticles on cardiac injury biomarkers in 5-fluorouracil–treated rats. (A) CK-MB, (B) cardiac troponin I (cTnI), and (C) LDH activities. The data, which includes statistical comparisons between groups, are presented as mean ± SEM (*n* = 5). *P < 0.05, **P < 0.01, ***P < 0.001, and ****P < 0.0001.

As presented in Figures 7, administration of 5-fluorouracil (5-FU) caused pronounced cardiovascular disease, as demonstrated by marked elevations in serum cardiac enzyme activities. Compared with the control group, 5-FU-treated rats exhibited substantial increases in CK-MB, troponin, and LDH by 77.17%, 106.25%, and 101.30%, respectively, indicating severe myocardial injury, disruption of cardiomyocyte membrane integrity, and enhanced leakage of intracellular enzymes into the circulation (*p* < 0.05).

Co-treatment with sodium selenite (Na₂SeO₃ & 5-FU) resulted in only partial amelioration of 5-FU-induced cardiac enzyme disturbances. Relative to the Normal Control group, CK-MB, troponin, and LDH levels remained elevated by 17.86%, 14.43%, and 35.27%, respectively. However, in contrast to the 5-FU group, these parameters showed moderate reductions of 33.50%, 44.53%, and 32.80%, reflecting a limited cardioprotective effect of inorganic selenium against 5-FU-induced myocardial damage (*p* < 0.05).

In contrast, kaempferol supplementation (KMP & 5-FU) produced a more pronounced protective effect on cardiac enzyme profiles. for the 5-FU group, serum CK-MB, troponin, and LDH activities were reduced by 38.43%, 37.04%, and 10.93%, respectively. Despite this improvement, enzyme levels remained greater than those observed in the Normal Control group, showing residual elevations of 9.07%, 29.82%, and 79.28%, suggesting partial but incomplete restoration of myocardial integrity (*p* < 0.05).

Notably, treatment with selenium nanoparticles biosynthesized using kaempferol (KMP-SeNPs & 5-FU) conferred the most substantial cardioprotective effect among all treated groups. In relation to the 5-FU group, CK-MB, troponin, and LDH concentrations were markedly reduced by 40.67%, 40.03%, and 26.56%, correspondingly. In contrast to the Normal Control group, enzyme activities exhibited only mild residual increases of 5.16%, 23.71%, and 47.81%, approaching near-physiological values and demonstrating superior preservation of myocardial structural and functional integrity (*p* < 0.05).

### Oxidative stress and redox homeostasis markers

**Fig. 8:**
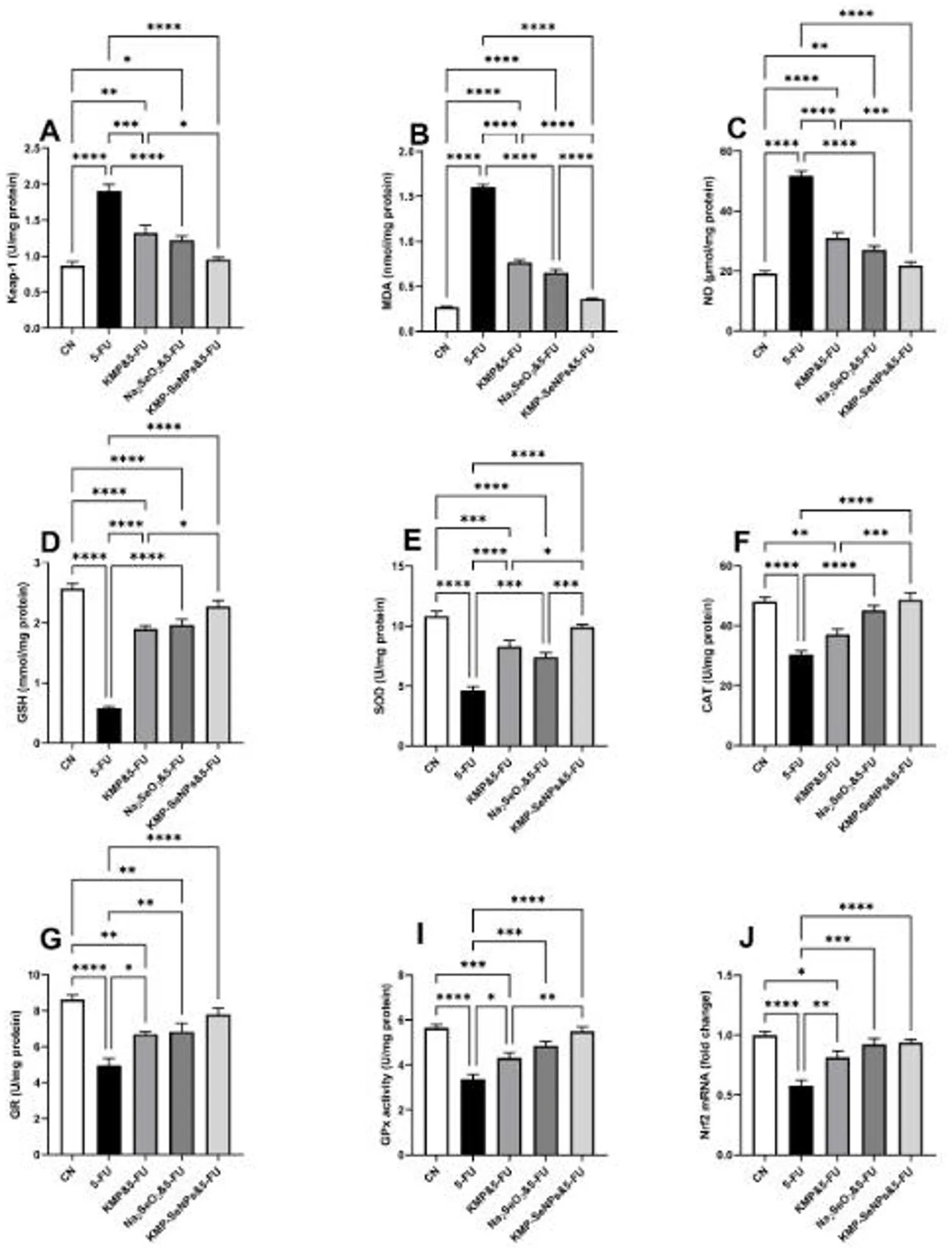
Effects of kaempferol and kaempferol-mediated selenium nanoparticles on oxidative stress and antioxidant defense in 5-fluorouracil–induced toxicity. (A) Keap-1, (B) MDA, (C) NO, (D) GSH, (E) SOD, (F) CAT, (G) GR, (I) GPx, and (J) Nrf2 expression. Data are presented as mean ± SEM (*n* = 5) with statistical comparisons among groups. *P < 0.05, **P < 0.01, ***P < 0.001, and ****P < 0.0001.

As presented in Figures 8, administration of 5-fluorouracil (5-FU) induced a profound oxidative stress state and severe disruption of redox homeostasis in cardiac tissue. Compared with the Normal Control group, 5-FU-treated rats exhibited marked elevations in Keap-1, malondialdehyde, and nitric oxide (NO) levels by 120.25%, 500.60%, and 170.67%, respectively. These changes were accompanied by pronounced suppression of endogenous antioxidant defenses, as evidenced by significant reductions in GSH, SOD, CAT, GR, GPx, and Nrf2 by 38.84%, 43.41%, 55.11%, 38.69%, 40.49%, and 41.82%, respectively, confirming excessive ROS and NOS production, antioxidant depletion, and inhibition of cytoprotective signaling pathways following 5-FU intoxication (*p* < 0.05).

Oral supplementation with inorganic selenium (Na₂SeO₃ & 5-FU) resulted in only partial mitigation of oxidative damage. Relative to the Normal Control group, Keap-1, MDA, and NO levels remained significantly elevated by 40.89%, 144.71%, and 41.29%, respectively, while antioxidant parameters including GSH, SOD, CAT, GR, GPx, and Nrf2 continued to show residual reductions of 20.34%, 20.06%, 27.56%, 16.89%, 14.11%, and 7.65%, respectively. When contrasted with the 5-FU group, these alterations reflected modest improvements in oxidative status, indicating a limited protective efficacy of inorganic selenium against 5-FU-induced redox imbalance (*p* < 0.05).

In contrast, kaempferol supplementation (KMP & 5-FU) exerted a more substantial antioxidative effect. Compared with the 5-FU group, Keap-1, MDA, and NO levels were significantly reduced by 30.75%, 52.18%, and 39.98%, respectively. Concurrently, GSH, SOD, CAT, GR, GPx, and Nrf2 levels were markedly restored by 45.16%, 44.53%, 66.23%, 31.42%, 28.60%, and 40.66%, respectively. Despite these improvements, most oxidative stress and antioxidant parameters did not fully return to Normal Control values, indicating partial but incomplete restoration of redox homeostasis (*p* < 0.05).

Notably treatment with selenium nanoparticles biosynthesized using kaempferol (KMP-SeNPs & 5-FU) produced the most pronounced normalization of oxidative stress biomarkers. Relative to the 5-FU group, Keap-1, MDA, and NO levels declined markedly by 50.05%, 77.60%, and 63.06%), respectively, approaching near-physiological values. Simultaneously, antioxidant defenses were robustly enhanced, with GSH, SOD, CAT, GR, GPx, and Nrf2 levels increasing by 75.28%, 76.12%, and 122.38%, 63.29%, 63.34%, and 62.02%), respectively. By comparing it with the Normal Control group.

### Inflammatory cytokines and immunoregulatory gene expression

**Fig. 9:**
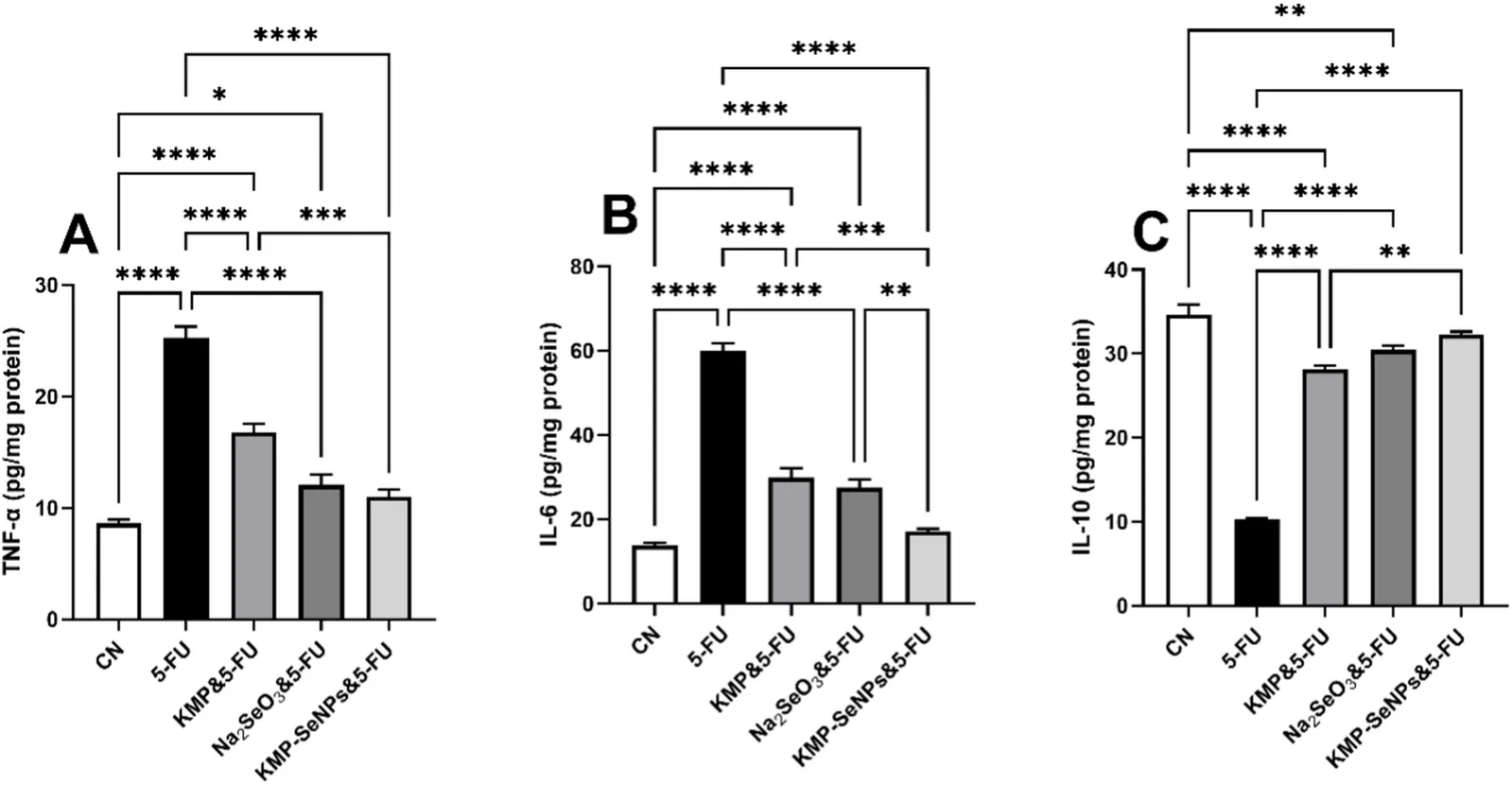
Effects of kaempferol and kaempferol-mediated selenium nanoparticles on inflammatory cytokines in 5-fluorouracil–induced toxicity. (A) TNF-α, (B) IL-6, and (C) IL-10 protein values. Data are presented as mean ± SEM (*n* = 5) with statistical comparisons among groups. *P < 0.05, **P < 0.01, ***P < 0.001, and ****P < 0.0001.

As illustrated in Figures 9, administration of 5-fluorouracil (5-FU) induced a marked inflammatory reaction accompanied by profound disruption of immune regulatory signaling. Compared with the Normal Control group, TNF-α and IL-6 values in 5-FU-administered rats escalated significantly by 192.95%, and 333.54%, respectively, while IL-10 was significantly reduced by 70.31%.

**Fig. 10:**
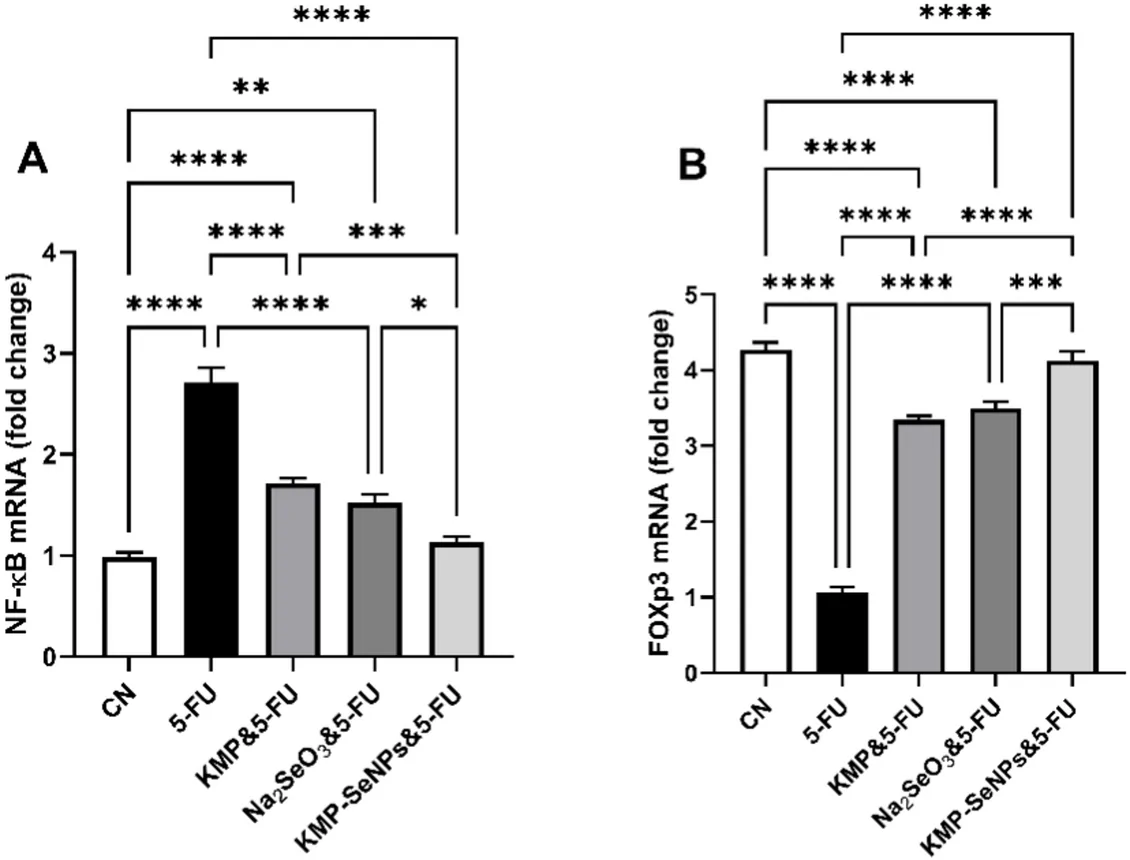
Effects of kaempferol and kaempferol-mediated selenium nanoparticles on gene expression in 5-fluorouracil–induced toxicity. mRNA expression of (A) NF-κB and (B) FOXP3. Data are presented as mean ± SEM (*n* = 5) with statistical comparisons among groups. *P < 0.05, **P < 0.01, ***P < 0.001, and ****P < 0.0001.

At the molecular level, expression of NF-κB was strongly upregulated by 175.45%, whereas expression of the regulatory T-cell marker FOXP3 was markedly downregulated by 75.08%. These findings indicate excessive inflammatory activation, cytokine imbalance, and impairment of immunoregulatory mechanisms following 5-FU exposure (*p* < 0.05) (Figs. 10**)**.

Co-treatment with sodium selenite (Na₂SeO₃ & 5-FU) resulted in partial improvement of inflammatory and transcriptional disturbances. Relative to control group, TNF-α and IL-6 values remained elevated by 39.90% and 99.17%, respectively, while IL-10 showed a modest reduction by 12.15%. NF-κB expression persisted above basal levels by 54.45%, and FOXP3 expression remained reduced by 18.07%. When evaluated against the 5-FU group, sodium selenite reduced TNF-α and IL-6 by 52.27% and 54.06%, respectively, increased IL-10 by 195.68%, suppressed NF-κB expression by 43.92%, and enhanced FOXP3 expression by 228.58%, indicating a moderate but incomplete anti-inflammatory and immunoregulatory effect (*p* < 0.05).

In contrast, kaempferol supplementation (KMP & 5-FU) produced a more pronounced attenuation of inflammatory signaling. In contrast to 5-FU group, TNF-α and IL-6 levels declined by 33.53%, and 50.17%, respectively, while IL-10 increased substantially by 173.23%. Concurrently, NF-κB expression was reduced by 36.82%, and FOXP3 expression was restored by 213.86%. However, relative to the control group, TNF-α, IL-6, and NF-κB remained elevated by 94.78%, 116.09%, and 73.99%, respectively, and FOXP3 expression remained reduced by 21.75%, reflecting partial recovery of inflammatory and immunoregulatory balance (*p* < 0.05).

Notably, treatment with selenium nanoparticles biosynthesized using kaempferol (KMP-SeNPs & 5-FU) exerted the most robust protective effect. Notably, the 5-FU group, TNF-α and IL-6 were markedly reduced by 56.45%, and 71.36%, respectively, while IL-10 increased by 213.27%. In parallel, NF-κB expression was strongly suppressed by 58.55%, and FOXP3 expression was markedly enhanced by 287.48%. Compared with the control group, TNF-α, IL-6, and NF-κB exhibited only minimal residual elevations by 27.59%, 24.13%, and 14.18%, respectively, while FOXP3 expression was nearly normalized by 3.41%, demonstrating near-complete restoration of inflammatory and immunoregulatory homeostasis (*p* < 0.05) **(**Figs. 9 and 10**)**.

### Apoptotic biomarkers

As illustrated in **Figures (**11**)**, administration of 5-FU triggered a pronounced apoptotic response in cardiac tissue, as reflected by marked alterations in key pro- and anti-apoptotic markers. In contrast to the control group, 5-FU-injected rats exhibited significant increases in Bax and Caspase-3 by 83.38%, and 215.22%, respectively, accompanied by a substantial reduction in Bcl-2 by 49.60%. These findings indicate excessive activation of apoptotic signaling pathways and loss of cellular survival mechanisms following 5-FU exposure (*p* < 0.05).

Co-treatment with sodium selenite (Na₂SeO₃ & 5-FU) resulted in partial attenuation of apoptosis-related disturbances. Relative to the control group, Bax and Caspase-3 levels remained elevated by 32.19%, and 45.33%, respectively, while Bcl-2 remained reduced by 15.57%. When compared with the 5-FU group, sodium selenite administration reduced Bax and Caspase-3 by 27.93%, and 53.90%, respectively, and increased Bcl-2 by 67.45%, indicating a moderate anti-apoptotic effect that was insufficient to fully restore apoptotic balance (*p* < 0.05).

In contrast, kaempferol supplementation (KMP & 5-FU) exerted a more pronounced modulatory effect on apoptotic signaling. Compared with the 5-FU group, Bax and Caspase-3 levels declined by 32.49%, and 49.38%, respectively, while Bcl-2 levels increased by 44.68%. However, relative to the control group, Bax and Caspase-3 remained elevated by 23.87%, and 59.54%, respectively, and Bcl-2 remained reduced by 27.04%, indicating partial but incomplete suppression of apoptosis (*p* < 0.05).

Notably, treatment with selenium nanoparticles biosynthesized using kaempferol (KMP-SeNPs & 5-FU) produced the most substantial anti-apoptotic effect. Relative to the 5-FU group, Bax and Caspase-3 levels were markedly reduced by 42.86%, and 66.59%, respectively, while Bcl-2 expression was significantly enhanced by 68.13%. Compared with the control group, Bax and Caspase-3 exhibited only minimal residual increases of 4.77%, and 5.27%, respectively, and Bcl-2 levels approached near-physiological values 15.23%, indicating near-complete restoration of apoptosis-regulatory balance and highlighting the superior cytoprotective efficacy of KMP-SeNPs against 5-FU-caused cardiac injury (*p* < 0.05).

**Fig. 11:**
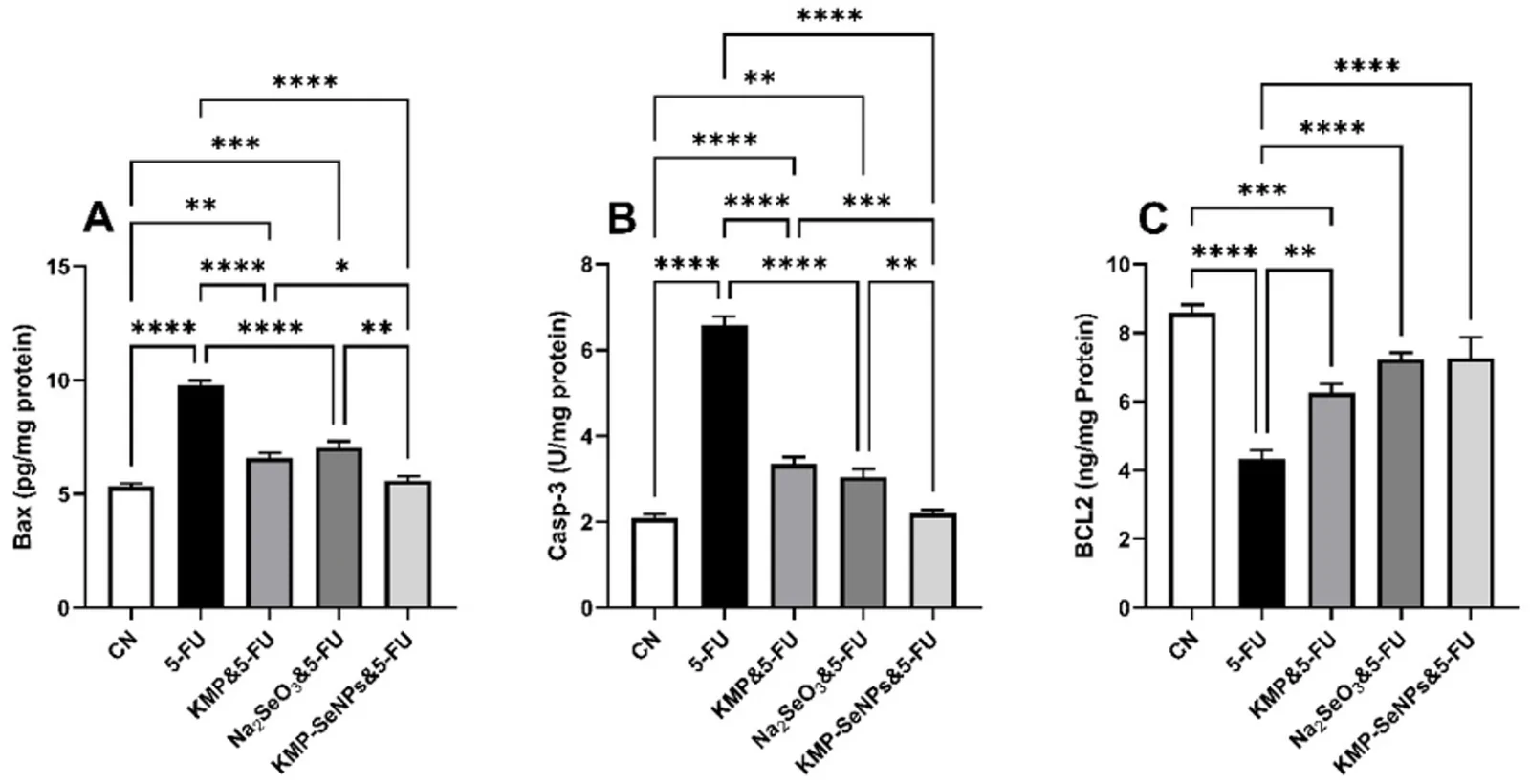
Effects of kaempferol and kaempferol-mediated selenium nanoparticles on apoptotic markers in 5-fluorouracil–induced toxicity. (A) Bax, (B) Caspase-3, and (C) Bcl-2 values in experimental groups. Data are presented as mean ± SEM (*n* = 5) with statistical comparisons among groups. *P < 0.05, **P < 0.01, ***P < 0.001, and ****P < 0.0001.

### Immunohistochemical examination of kaempferol-mediated selenium nanoparticles on Nrf-2 and Keap-1

**Fig. 12:**
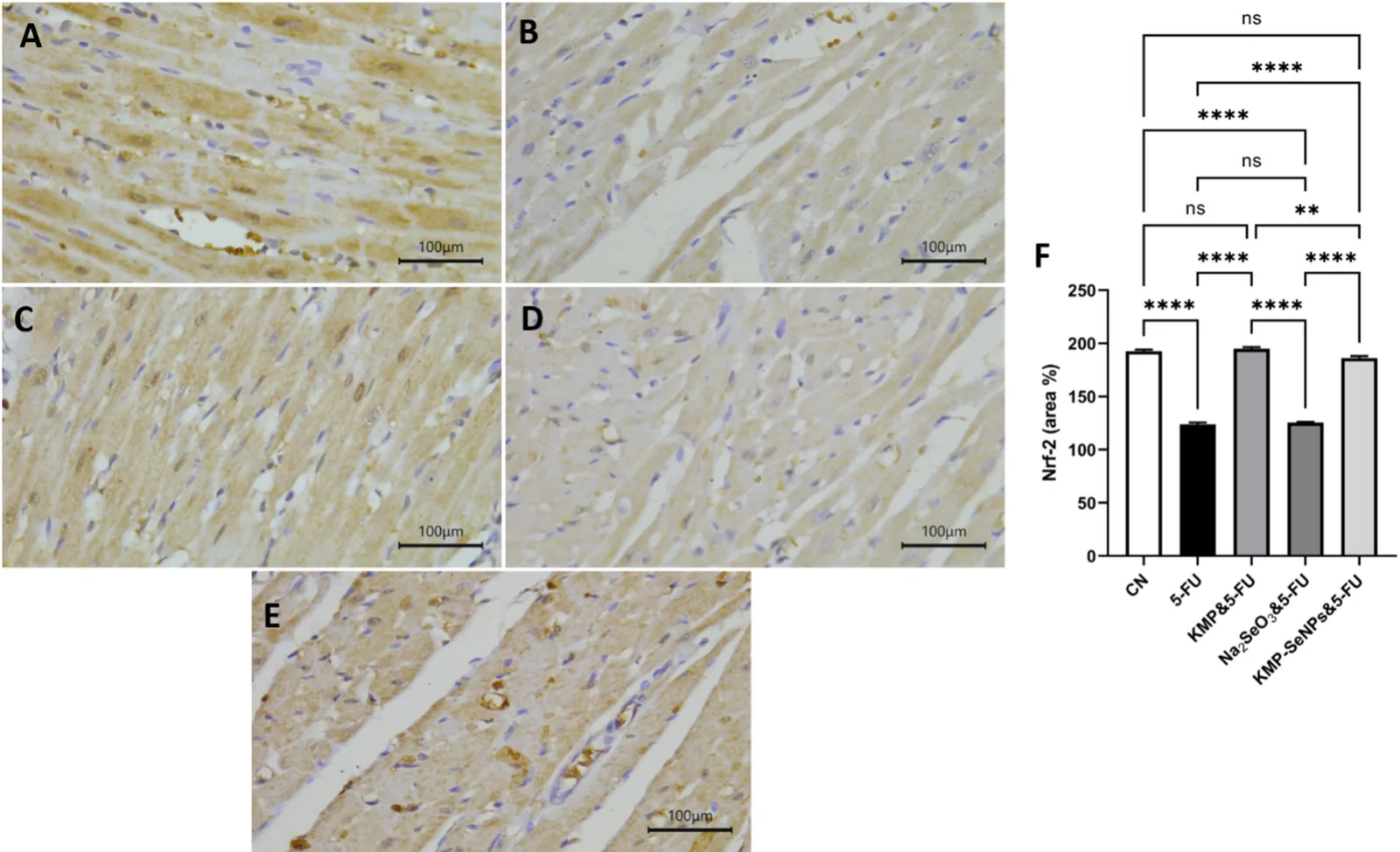
Protective effect of KMP-SeNPs on cardiac immunoreactivity of Nrf-2 in 5FU-induced cardiotoxicity in rats. Photomicrographs of tissue of the heart from all cohorts. The reactivity of Nrf-2 was evident in the tissues as a brown tone produced by DAB chromogen. (DAB, ×400). (A) The control group showed normal cardiac muscle structure with moderate Nrf-2 staining. (B) 5FU and (D) Na_2_SeO_3_&5FU treated groups revealed weak Nrf-2 immunostaining. In contrast, (C) KMP&5FU group revealed moderate expression, while (E) KMP-SeNP&5FU-treated group displayed moderate Nrf-2 staining. (F) Quantitative assessment of immunostaining region % for Nrf-2 was expressed as mean ± SEM (*n* = 5). Statistical evaluation utilizing one-way ANOVA accompanied by Duncan’s post hoc analysis. ns: not significant; *P < 0.05, **P < 0.01, ***P < 0.001, and ****P < 0.0001.

IHC analysis revealed a marked expression of the NRF-2 in cardiac tissue of control, KMP-SeNPs&5FU, and KMP-5FU treated groups without prominent variance (*p* > 0.05), whereas the Na_2_SeO_3_&5FU and 5FU groups had weak expression. Both KMP&5FU group and KMP-SeNPs&5FU groups differed significantly with 5FU regarding Nrf-2 IHC immunoreactivity (*P* < 0.001) **(**Fig. 12**)**.

**Fig. 13:**
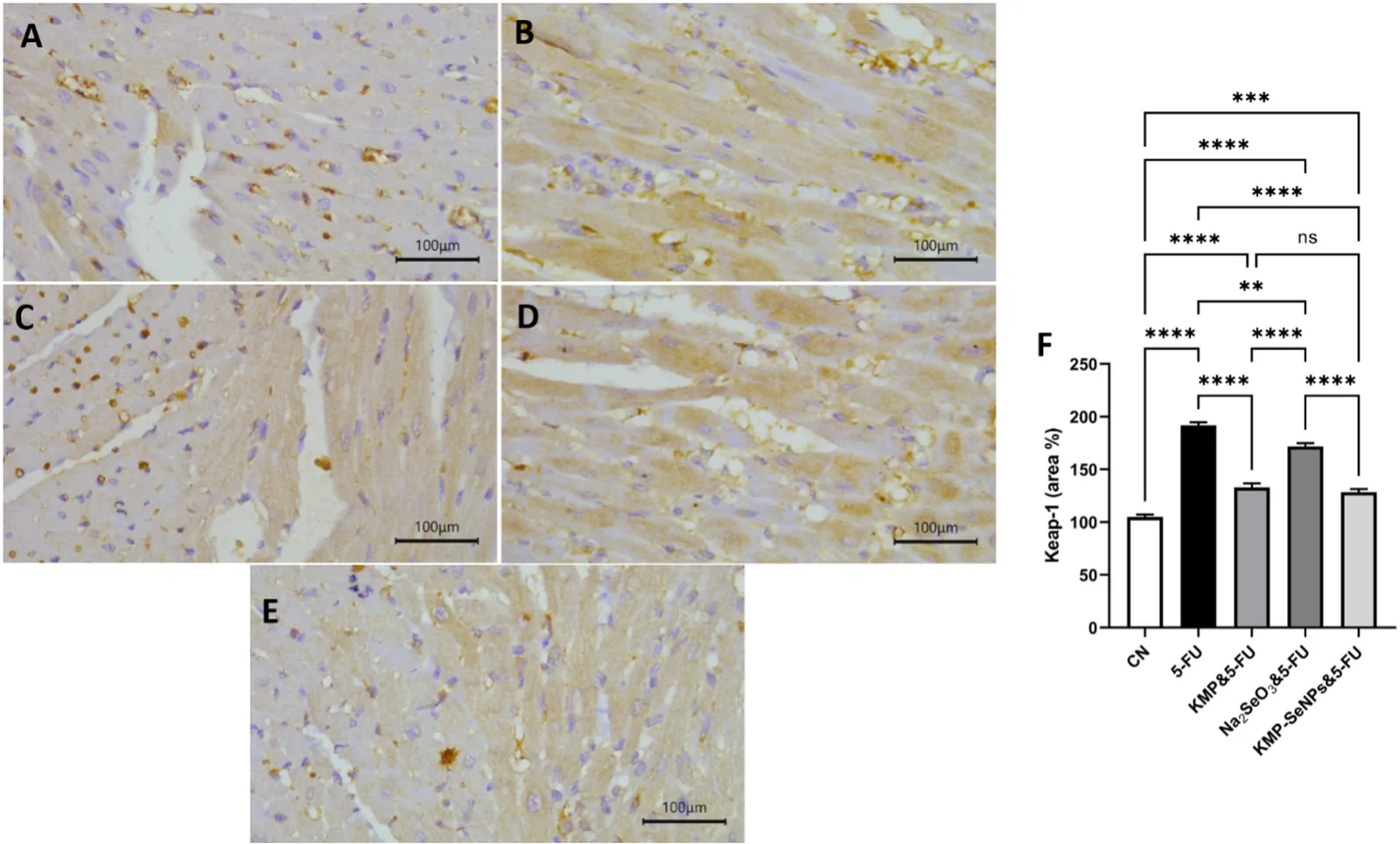
Protective effect of KMP-SeNPs on cardiac immunoreactivity of Keap-1 in 5FU-induced cardiotoxicity in rats. Photomicrographs of cardiac tissues from all groups. The eactivity of Keap-1 was observed in the tissues as a brown hue generated by the DAB chromogen. (DAB, ×400). (A) The control group showed normal cardiac muscle structure with weak Keap-1 staining. (B) 5FU and treated groups revealed marked Keap-1 immunostaining. In contrast, (D) Na_2_SeO_3_&5FU group revealed moderate expression, while (C) KMP&5FU and (E) KMP-SeNP&5FU-treated group displayed mild Keap-1 staining. (F) Quantitative assessment of immunostaining region % for Keap-1 was expressed as mean ± SEM (*n* = 5). Statistical analysis by one-way ANOVA with Duncan’s post hoc test. ns: non-significant; *P < 0.05, **P < 0.01, ***P < 0.001, and ****P < 0.0001.

Moreover, Keap1 immunohistochemical staining was mild in the cardiac tissues of the control, KMP-SeNPs&5FU, and KMP&5FU groups. Although both treatment groups exhibited slightly higher Keap1 expression than the control group, the difference was statistically significant. In contrast, the Na2SeO3&5-FU showed moderate expression and 5-FU groups showed moderate Keap1 immunoreactivity (Fig. 13). Furthermore, the KMP-SeNPs&5FU group exhibited significantly lower Keap1 immunoreactivity than the 5FU group (*P* < 0.001).

### Histopathological examination

The histological analysis of the control group demonstrated typical heart tissue architecture, including branched muscle fibers with central oval vesicular nuclei, acidophilic sarcoplasm, and extracellular gaps with blood capillaries **Figure (**14** A).** The 5FU group revealed symptoms of cardiac necrosis, including substantial fragmentation, hypereosinophilia, perimysial edema, and nuclear pyknosis. The interstitial oedema in the tissue gaps increased significantly **Figure (**14**B).** Na_2_SeO_3_−5FU treated group showed limited improvement compared to 5FU group with pyknotic myocytes and perimysial edema **Figure (**14** C).** The morphological appearance in the KMP&5FU-treated group was significantly improved, with myocytic morphology preserved and interstitial capillaries eliminated **Figure (**14**D).** The KMP-SeNPs&5FU-treated group displayed an approximately normal histology pattern of cardiac myocytes **Figure (**14**E).**

**Fig. 14:**
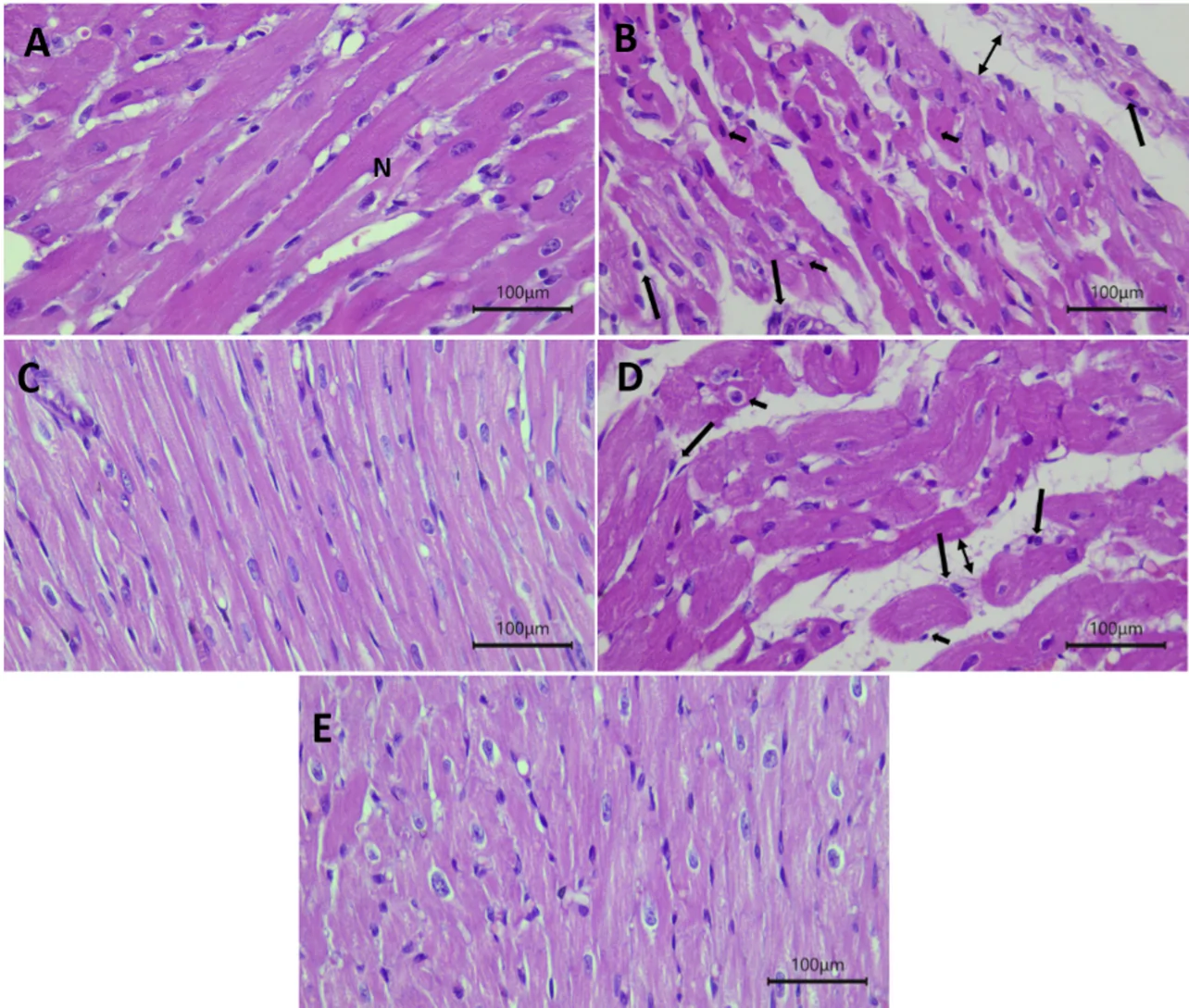
Histopathological analysis of cardiac tissues. (A) Photomicrograph of the cardiac tissue of control rat showing normal histological structure cardiac myocytes with acidophilic myocardial fibers, oval vesicular nuclei (N). (B) Section from 5FU-treated group showed necrotic muscle fibers (*) with pyknotic cardiomyocytes (short arrow), widely separated myocytes (double arrow) and inflammatory cellular infiltration (long arrow). (D) Na_2_SeO_3_&5FU group showed perimysial edema (double arrow) and inflammatory cellular infiltration (long arrow), and apoptotic cell (short arrow). Both (C) KMP&5FU and (E) KMP-SeNPs&5FU-treated group showed preserved cardiomyocyte morphology with central oval vesicular nuclei (N), exhibiting approximately normal structure. (H&E, ×400).

## Discussion

**Fig. 15:**
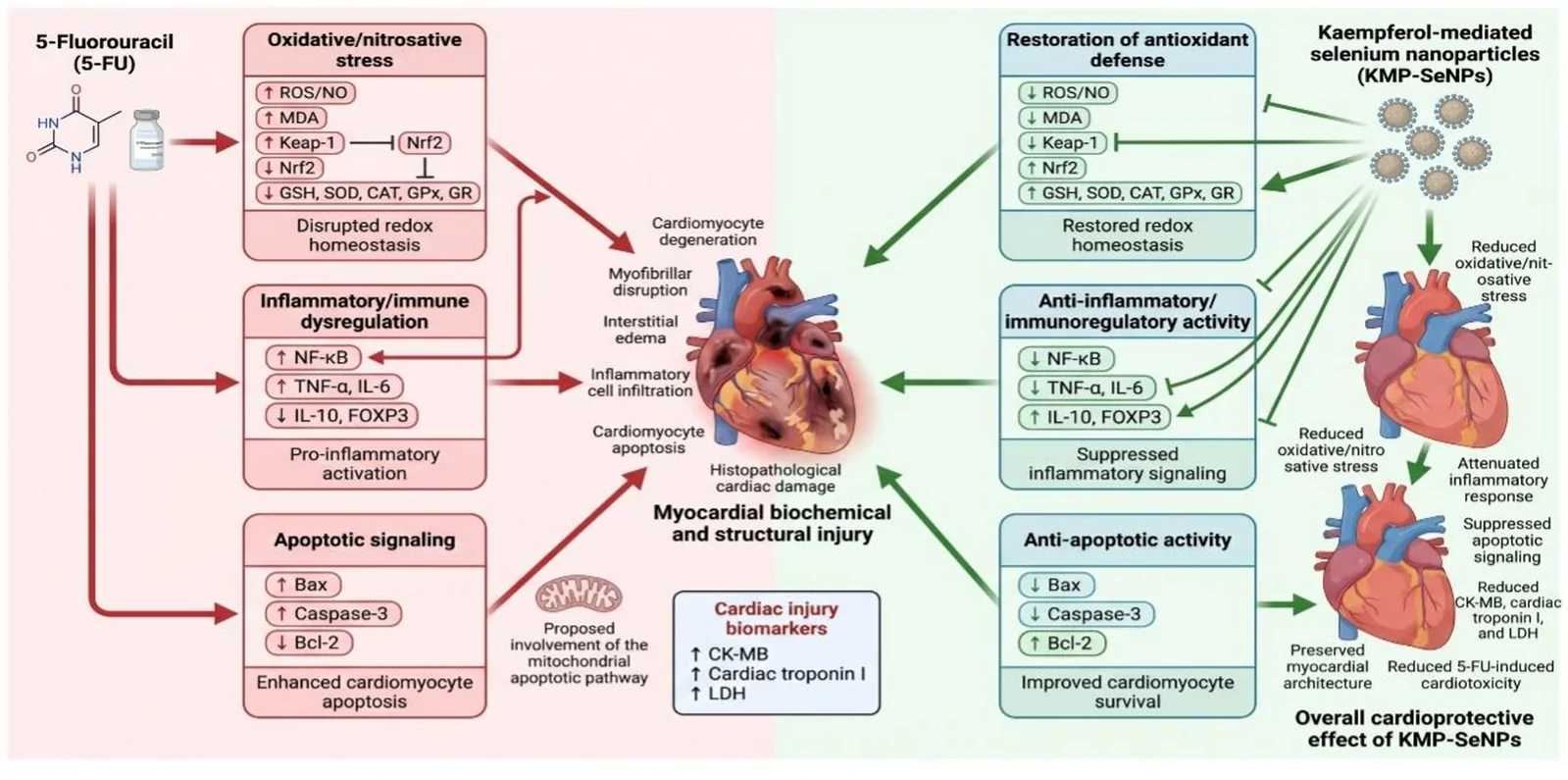
Proposed mechanistic model of the cardioprotective effects of kaempferol-mediated selenium nanoparticles (KMP-SeNPs) against 5-fluorouracil-induced cardiotoxicity, (generated using biorender).

The present study investigated the cardioprotective efficacy of kaempferol-mediated selenium nanoparticles against 5-fluorouracil-induced cardiac injury by integrating biochemical, molecular, immunohistochemical, and histopathological assessments. Exposure to 5-FU produced marked myocardial damage characterized by elevated cardiac injury biomarkers, disrupted redox homeostasis, activation of NF-κB-mediated inflammation, impaired immunoregulatory signaling, and enhanced apoptotic responses. Although free kaempferol and sodium selenite provided partial protection, KMP-SeNPs exerted the most pronounced beneficial effects by restoring the Nrf2/Keap-1 antioxidant axis, suppressing pro-inflammatory mediators, enhancing IL-10 and FOXP3 expression, and rebalancing Bax/Bcl-2/caspase-3 signaling. These coordinated effects were accompanied by substantial preservation of myocardial architecture, supporting the superior cardioprotective potential of the nanoformulation against 5-FU-induced cardiotoxicity as shown in Fig. 15.

The 5-FU is still considered to be one of the most common antimetabolites chemotherapy drugs used as an agent in the treatment of wide variety of solid tumors. The clinical utility of 5-FU is often limited by dose-limiting toxicities of the important body organs, especially the cardiovascular system, despite its clinical efficacy^25^. The 5-FU-induced cardiotoxicity is ranked as the second most commonly occurring type of chemotherapy-induced cardiac injury after anthracyclines^26^. The pathogenesis of this cardiotoxicity is complicated and multifaceted as it includes direct myocardial damage, dysfunction of endothelia, inflammation, impairment of mitochondria, and programmed cell death. Oxidative stress is a key stimulating and reinforcing factor in heart tissue damage due to 5-FU despite accumulating experimental and clinical evidence^27^.

Occurrences of OS when the production of the concentration of ROS and NOS exceeds the capacity of the endogenous antioxidant defensive systems^5^. Oxidative injury affects cardiomyocytes especially because of their dense mitochondrial distribution and the constant energy requirement^28^. This experiment, 5-FU treatment led to the development of an intense oxidative imbalance, which was manifested by high levels of malondialdehyde and reduced glutathione (GSH), and significant reductions in the activity of important antioxidant enzymes, including GPx, SOD, and CAT. MDA is a trustworthy biomarker of oxidative injury of the membrane^29^. The presence of the increased MDA is indicative of the significant peroxidative destruction of membrane lipids, which undermines the integrity of cell membranes and cellular activity of the cardiomyocytes^6^, as shown in Fig. 15.

The molecular components behind the oxidative damage triggered by 5-FU were highly linked with impairment of Nrf2 signaling pathway. Nrf2 along with its cytoplasmic suppressor Keap1 is a regulatory master system that controls the cellular redox homeostasis, detoxification of cellular functions and modulation of inflammation^25^. In physiological conditions, Nrf2 coordinates the expression of antioxidant enzyme, Phase I and Phase II detoxification enzyme and cytoprotective agent genes. Nrf2 is extremely important in myocardial resilience to oxidative and metabolic stress, tissue repair, and maladaptive remodeling in cardiac tissue^30^.

In the present research, the exposure of 5-FU greatly reduced the Nrf2 expression and increased the Keap1 expression, thereby inhibiting the antioxidant genes expression and oxidative injury worsening^31^. This sort of Nrf2/Keap1 axis dysfunction makes cardiomyocytes very susceptible to oxidative damage and assists in the secondary modulating of inflammatory and apoptotic mediators. The results of these studies are consistent with earlier studies that confirmed that one of the main molecular factors in chemotherapy-induced heart injury is the inhibition of Nrf2^32^.

Kaempferol is a natural flavonoid that is highly abundant in numerous fruits and vegetables and is commonly characterized by a high level of antioxidant, anti-inflammatory, and cardioprotective effects^33^. The administration of kaempferol in the current study partially reduced OS induced by 5-FU as indicated by a lower level of MDA and greater content and antioxidant enzyme activity^34^. These repercussions are in line with the ability of kaempferol to scavenge reactive oxygen species, as well as the ability to regulate redox-sensitive signaling. Kaempferol has been reported to act mechanistically, by inducing Nrf2 activation by disrupting Keap1-mediated degradation, which increases the transcription of antioxidant defense genes^35^.

Regardless of these positive influences, kaempferol was not sufficient in its effect to achieve the restoration of redox balance and avoid myocardial structural damage^7,36^. This partial protection can be explained by the disadvantages of free kaempferol such as low aqueous solubility, fast metabolism, and low intracellular bioavailability^37^.

Selenium is an important trace mineral, which has far-reaching antioxidant properties because of its incorporation into selenoproteins^38,39^. These enzymes assume vital functions in countering peroxides, redoxing, and protecting biological components against oxidative damages^40,41^. The administration of sodium selenite in this study increased the activities of antioxidant enzymes and decreased the lipid peroxidation, which validated the sodium selenite as a means of strengthening the endogenous antioxidant capacity. Selenium supplementation was also effective to partially recover Nrf2 expression and decrease levels of Keap1 which is an indication of redox-regulatory signaling^42^.

Nonetheless, selective selenium did not offer much cardioprotection. Although it had a strong ability to enhance enzymatic antioxidant defense, the effect on upstream signaling pathways, inflammatory regulation and the control of apoptosis was still small^43^. Moreover, inorganic selenium compounds have a wide therapeutic index, which can restrict their usability in clinical use at certain doses required to produce a strong cardioprotective effect^44^.

The best observation of the current research is the better cardioprotective activity of the selenium nanoparticles biosynthesized with kaempferol (KMP-SeNPs). In all the parameters analyzed, KMP-SeNPs significantly improved compared to free kaempferol and sodium selenite, which suggests that the nanoparticle formulation contributes to therapeutic efficacy significantly^45^. This high effectiveness is directly associated with the effective preparation and physicochemical optimization of the nanoformulation^46^.

The green strategy used in the preparation of KMP-SeNPs allowed to reduce the amount of Se ions and stabilize NPs at the same time with the help of kaempferol as reducing and stabilizing reagent^10^. This biocompatible and translational approach does not use any toxic reagents or any harsh conditions, which increases environmental friendliness and biocompatibility^47^. The results of the physicochemical characterization showed the production of selenium nanoparticles of stable size and good surface charge properties^43^. The small size of nanoscale enables cell uptake and uptake into the myocardial tissue, and the negative surface charge is important to provide colloidal stability and evade aggregation when in physiological environments^48^.

Transmission electron microscopy showed mainly spherical and well-spread nanoparticles, which means that kaempferol was successfully functionalized on the surface^49^. Notably, kaempferol coating the surface of the nanoparticle is an active biointerface that can directly engage with intracellular signaling pathways^44^. Such two-dimensional properties encompass selenium-mediated enzymatic antioxidant support and kaempferol-mediated signaling modulation, which gives a mechanistic explanation of the superior cardioprotective effect with KMP-SeNPs^33,46^.

On a mechanistic level, the intervention of KMP-SeNP led to the establishment of almost complete recovery of redox homeostasis with the intensive activation of the Nrf2 pathway and suppressing the expression of Keap1^50^. This was coupled with the normalization of GSH levels and antioxidant enzyme activities which have been effective in alleviating oxidative and nitrosative stress. KMP-SeNPs was found to have a more sustained and comprehensive activation of intrinsic antioxidant mechanisms compared to free kaempferol or selenium, which indicates the relevance of nanoparticle-mediated delivery overcoming bioavailability barriers^33,51^.

Another very important element of cardiotoxicity caused by 5-FU is inflammation. NF-κB is one of the key transcription factors that regulate inflammation, production of cytokines and cellular survival^52^. NF-κB activation enhances the expression of Pro-inflammatory cytokines, such as TNF-α and IL- 10, to overcome inflammation and functional loss in the heart^53^. In this paper, 5-FU significantly stimulated NF-κB signaling, enhanced the levels of pro-inflammatory cytokines and suppressed IL-10^42^. These inflammatory changes were confirmed by histopathology that enabled the inflammatory cell infiltration in cardiac tissue.

Inflammatory responses were partially suppressed by kaempferol and sodium selenite interventions that inhibited the NF-κB signal transduction and reduced the levels of pro-inflammatory cytokine levels. Nevertheless, the strongest anti-inflammatory effects were achieved with KMP-SeNPs^54^, who significantly inhibited the activity of NF- 2 and IL-10 expression and suppressed the expression of downstream mediators. This increased anti-inflammatory effect can be explained by the long-term Nrf2-activation because Nrf2 is reported to inhibit inflammatory crosstalk via NF-κB^55^.

There is a significant role of apoptosis in the development of 5-FU–induced cardiotoxicity, especially in the cases of prolonged oxidative stress and inflammation^7^. The present study revealed that 5-FU significantly up-regulated the executioner caspase-3 and down-regulated the expression of the anti-apoptotic protein Bcl-2, which is an indication of mitochondrial apoptotic pathways activation^27,56^. The anti-apoptotic effects partially attenuated these apoptotic alterations by kaempferol and selenium, but KMP-SeNPs showed the highest anti-apoptotic effect. When using KMP-SeNPs, the activation of caspase-3 and the restoration of Bcl-2 levels were inhibited, which suggests that mitochondrial integrity should be stabilized and cardiomyocyte apoptosis should be prevented^32^.

The present study has several limitations. Only a single dose of kaempferol, sodium selenite, and KMP-SeNPs was evaluated, precluding assessment of dose–response relationships and long-term safety. The relatively short experimental duration also did not permit investigation of chronic or delayed cardiotoxicity associated with either 5-FU or KMP-SeNPs. Moreover, cardiac protection was assessed mainly through biochemical, molecular, immunohistochemical, and histopathological endpoints, without direct functional evaluation by electrocardiography or cardiac imaging. In vitro cytoprotective validation using cardiomyocyte models, such as H9c2 cells, was also not performed before the in vivo study. Future investigations should therefore include cell-based mechanistic assays, ECG and echocardiographic assessments, multiple dosing regimens, prolonged follow-up, and comprehensive safety evaluation to further confirm the functional and translational cardioprotective potential of KMP-SeNPs.

## Conclusion

The current study has shown that selenium nanoparticles kaempferol functionalized (KMP-SeNPs) offer potent protection against cardiotoxicity caused by 5-fluorouracil in a combination of antioxidant, anti-inflammatory, immunoregulatory as well as anti-apoptotic actions. The biological relevance of kaempferol was confirmed by in silico docking that has shown a high affinity between kaempferol and several molecular targets including iNOS, TNF-α and GST, which are important molecular targets in oxidative stress and inflammatory signaling.

## Data Availability

Data will be made available on request.

## Abbreviations

KMP: Kaempferol
5-FU: 5-Flourouracil
SeNPs: Selenium nanoparticles
i.p.: Intraperitoneal
ROS: Reactive oxygen species
OS: Oxidative stress
BCL-2: B-cell lymphoma-2
Caspase-3: Cysteine aspartate-specific protease 3
DQC: Dimerization Quality Control
Keap-1: Kelch-like ECH-associated protein 1
Nrf2: Nuclear factor erythroid 2-related factor 2
DLS: Dynamic light scattering
TEM: Transmission Electron Microscopy
FT-IR: Fourier Transform Infrared
MDA: Malondialdehyde
LPO: Lipid peroxidation
NO: Nitroxide
GSH: Glutathione synthetase
SOD: Superoxide dismutase
CAT: Catalase
TNF-α: Tumor necrosis factor alpha
NF-κB: Nuclear factor kappa B
IL-1β: Interlukin-1β
Bax: Bcl-2 associated X-protein
GPx: Glutathione peroxidase
PDB: Protein Data Bank
FOXP3: Forkhead box P3
DAB: 3,3′-diaminobenzidine
iNOS: Inducible nitric oxide synthase
ELISA: Enzyme-Linked Immunosorbent Assay
qRT-PCR: Quantitative reverse transcription polymerase chain reaction
IHC: Immunohistochemistry
SEM: Standard Error of the Mean
